# Transcriptional slippage in the positive-sense RNA virus family *Potyviridae*

**DOI:** 10.15252/embr.201540509

**Published:** 2015-06-25

**Authors:** Allan Olspert, Betty Y-W Chung, John F Atkins, John P Carr, Andrew E Firth

**Affiliations:** 1 Division of Virology, Department of Pathology, Addenbrooke’s Hospital, University of CambridgeCambridge, UK; 2 Department of Plant Sciences, University of CambridgeCambridge, UK; 3 Schools of Biochemistry and Microbiology, University College CorkCork, Ireland; 4 Department of Human Genetics, University of UtahSalt Lake City, UT, USA

**Keywords:** gene expression, P3N-PIPO, Potyvirus, RNA virus, transcriptional slippage

## Abstract

The family *Potyviridae* encompasses ∼30% of plant viruses and is responsible for significant economic losses worldwide. Recently, a small overlapping coding sequence, termed *pipo*, was found to be conserved in the genomes of all potyvirids. PIPO is expressed as part of a frameshift protein, P3N-PIPO, which is essential for virus cell-to-cell movement. However, the frameshift expression mechanism has hitherto remained unknown. Here, we demonstrate that transcriptional slippage, specific to the viral RNA polymerase, results in a population of transcripts with an additional “A” inserted within a highly conserved GAAAAAA sequence, thus enabling expression of P3N-PIPO. The slippage efficiency is ∼2% in *Turnip mosaic virus* and slippage is inhibited by mutations in the GAAAAAA sequence. While utilization of transcriptional slippage is well known in negative-sense RNA viruses such as Ebola*,* mumps and measles, to our knowledge this is the first report of its widespread utilization for gene expression in positive-sense RNA viruses.

See also: **KA White** (August 2015)

## Introduction

**Figure 1:**
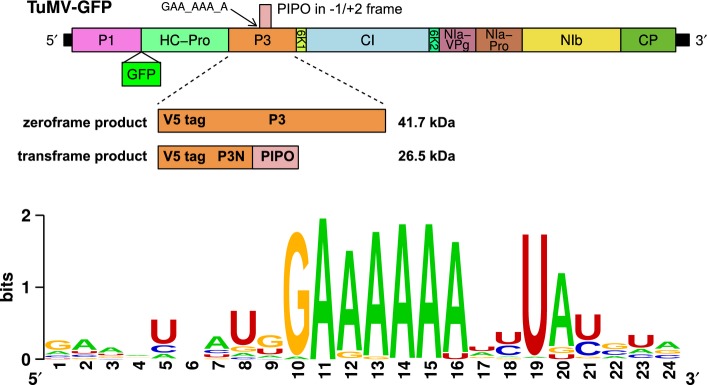
Schematic of the TuMV genome GFP is inserted between P1 and HC-Pro in the parent infectious clone (TuMV-GFP). Additionally, a V5 tag was inserted near the start of P3 to facilitate simultaneous detection of both P3 (zeroframe product) and P3N-PIPO (transframe product). The position of the conserved GAA_AAA_A sequence at the 5′ end of the *pipo* ORF is indicated. A WebLogo 56 representation of sequence conservation around the 5′ end of the *pipo* ORF for 99 genus *Potyvirus* NCBI RefSeqs aligned by amino acid sequence (see Appendix Dataset S1) is shown below.

The family *Potyviridae* encompasses around 30% of known plant virus species and causes more than half of viral crop damage worldwide 1,2. The family comprises the genera *Potyvirus*, *Rymovirus*, *Bymovirus*, *Ipomovirus*, *Tritimovirus*, *Macluravirus*, *Poacevirus* and *Brambyvirus*, with genus *Potyvirus* containing the most species. Family members have single-stranded monopartite positive-sense RNA genomes except in the genus *Bymovirus* where the genome is bipartite. The genomic RNA has a covalently linked 5′-terminal protein (VPg) and a 3′ poly(A) tail. Subgenomic transcripts are not produced 3. Until recently, all the viral proteins were thought to be encoded within a single open reading frame (ORF) (or one ORF per segment in bymoviruses) that is translated as a polyprotein and cleaved to produce the mature virus proteins. However, it is now thought that all potyvirids contain an additional coding ORF, termed *pipo*, that overlaps the P3-encoding region of the polyprotein ORF in the −1/+2 reading frame (Fig1) 4. PIPO is expressed as part of a larger product that was hypothesized to comprise the N-terminal part of P3 (termed P3N) fused to PIPO via either translational or transcriptional frameshifting 4. This hypothesis was further supported by the detection of products of appropriate sizes for P3 and P3N-PIPO with antibodies to N-terminal epitopes in P3 5,6. Frameshifting was proposed to occur at a GAA_AAA_A sequence (underscores separate polyprotein-frame codons) at the 5′ end of the *pipo* ORF that is highly conserved among *Potyvirus* species (Fig1). Members of other *Potyviridae* genera have similar homopolymeric runs of “A”s at the same site (Appendix Dataset S1). The frameshift product P3N-PIPO plays an essential role in cell-to-cell movement, and mutations within this motif result in a movement-deficient phenotype 5-7,8,9.

Many viruses utilize programmed ribosomal frameshifting (PRF) to direct a proportion of ribosomes into an alternative reading frame. In eukaryotic systems, efficient −1 PRF normally requires a “slippery” heptanucleotide sequence where the shift in reading frame takes place, and a 3′-adjacent stimulatory element which normally comprises an RNA stem-loop or pseudoknot structure separated from the slippery heptanucleotide by a “spacer” region of 5–9 nt 10,11. The consensus motif for the slippery heptanucleotide is X_XXY_YYZ, where XXX normally represents any three identical nucleotides; YYY represents AAA or UUU; Z represents A, C or U; and spaces separate zero-frame codons 12. In the tandem slippage model, the P-site anticodon re-pairs from XXY to XXX, whereas the A-site anticodon re-pairs from YYZ to YYY, allowing for perfect re-pairing except at the wobble position 13. Because the codon:anticodon duplex in the P site is not monitored so strictly as that in the A site, certain deviations from the canonical XXX of the slippery site are tolerated, including GGU, GUU, GGA and GAA 10-14. Frameshifting efficiency ranges from around 5–50%, depending on the particular system. In the absence of a 3′ stimulatory RNA structure, certain sequences can still support frameshifting to a level of potentially up to around 2% 12.

In general, RNA structures typical of −1 PRF stimulatory elements are not predicted to form at an appropriate spacing downstream of the potyvirus GAA_AAA_A sequence. Nonetheless, this could still be consistent with −1 PRF if only a very low level of frameshifting were required, or if there were atypical stimulatory elements (e.g. nascent peptide, mRNA–rRNA interactions, RNA structure involving base-pairing with distal elements in the genome or *trans*-acting factors 15-17,18). More importantly, however, the GAA_AAA_A sequence is in a different frame from the −1 PRF X_XXY_YYZ shift site motif, making it inconsistent with the tandem −1 slippage model. On the other hand, around half of *Potyvirus* species have a “G” preceding the conserved GAA_AAA_A sequence (making a canonical −1 PRF shift site G_GAA_AAA), while one might propose that other species use −1 PRF but with little re-pairing in the P site.

An alternative explanation is that frameshifting occurs at the transcriptional level. In several single-stranded negative-sense RNA viruses, such as members of the genus *Ebolavirus* and the sub-family *Paramyxovirinae*, the viral polymerase can stutter at a defined site to insert one or more additional nucleotides into a proportion of mRNA transcripts 19. Stuttering involves realignment between the template and nascent RNA strands in the polymerase and preferentially occurs on homopolymeric runs, especially those comprising “A”s or “U”s. In the paramyxoviruses, stuttering occurs on a 3′-U_n_C_m_-5′ (*n *+ *m *≥* *8) motif in the negative-sense template and results in the insertion of one or more additional “G”s in the positive-sense (5′-A_n_G_m_-3′) mRNA for the phosphoprotein gene. In paramyxoviruses, directionality is provided by the ability of RNA to form G:U pairs, but not A:C pairs. In ebolaviruses, stuttering occurs on a run of 7 “U”s in the negative-sense template to insert one or more additional “A”s in the positive-sense mRNA for the glyco-protein gene. However, transcriptional slippage was not considered an obvious explanation for *pipo* expression because of the short length of the conserved homopolymeric run (just 6 “A”s), and because the conserved 5′ “G” (resulting in a 3′-CU_6_-5′ sequence in the negative-sense template, opposite in orientation to the paramyxovirus 3′-U_n_C_m_-5′ stuttering site) appeared to favour nucleotide deletions over insertions, and two deletions (or one insertion) would be required to provide access to the *pipo* ORF.

To resolve the conundrum of *pipo* expression, we engineered an infectious *Turnip mosaic virus* (TuMV; genus *Potyvirus*) clone to express epitope-tagged P3/P3N-PIPO and used it to assess frameshift-ing efficiency in the natural context of virus infection. We performed mutational analyses of the GAA_AAA_A sequence and investigated the mutant phenotypes. Finally, we performed high-throughput sequencing of RNA derived from virus infections. We found that an extra “A” is inserted into the GA_6_ sequence in approximately 2% of TuMV transcripts, thus enabling expression of P3N-PIPO. Comparable editing frequencies (0.8–1.3%) were observed for two other potyviruses.

## Results and Discussion

### P3N-PIPO is expressed at very low levels in TuMV-infected plants

**Figure 2:**
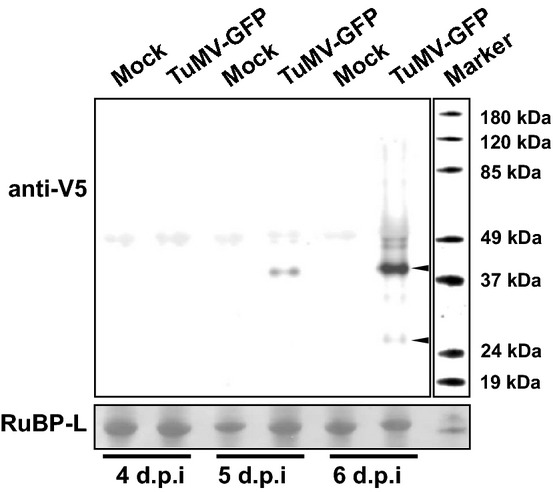
Detection of V5-tagged P3 and P3N-PIPO Total protein extracts from upper leaves of *Nicotiana benthamiana* plants agroinfiltrated with TuMV-GFP or mock-infiltrated were collected 4–6 d.p.i. Proteins were separated by SDS–PAGE, blotted and probed with V5 antibody. Bands corresponding to the theoretical size of tagged P3 (41.7 kDa) and P3N-PIPO (26.5 kDa) are indicated with arrowheads, visible in the “infected” lane at 6 d.p.i. Ponceau S staining of nitrocellulose membrane-bound RuBisCO large subunit (RuBP-L) was used as a loading control.

**Figure 3:**
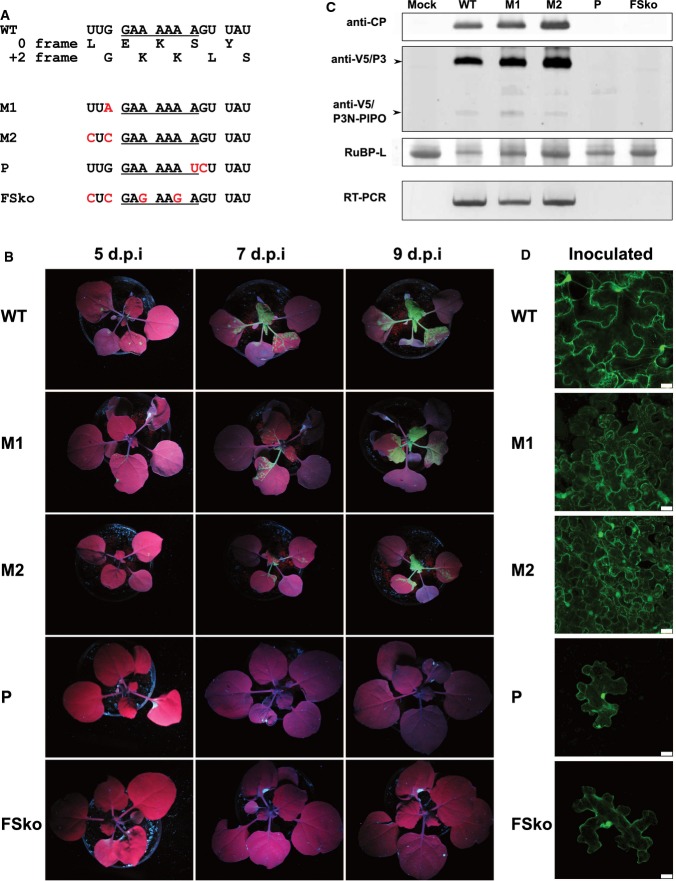
Analysis of TuMV mutants Sequences of slip site mutants. The highly conserved GAA_AAA_A (underlined) and flanking sequence in TuMV is shown at the top (WT). Spaces separate P3-frame codons. Theoretical translations in the 0 and −1/+2 frames are shown. Slip site mutants M1, M2, P and FSko are shown below with mutated nucleotides shown in red. All mutations leave the P3 amino acid sequence unaltered.

To investigate the potyvirus frameshifting mechanism, we used a GFP-expressing infectious clone of TuMV (TuMV-GFP; Fig1). To enable efficient detection of both P3 and P3N-PIPO with the same antibody, we inserted a sequence encoding the V5 epitope to tag both the P3 and P3N-PIPO proteins near to their N-termini (Fig1). The V5 tag was stably maintained within the virus genome for at least four passages. Using a V5 antibody, it was possible to detect both P3 and P3N-PIPO in protein extracts from upper leaves of plants following inoculation of lower leaves with TuMV-GFP via agroinfiltration (Fig2). P3 was detectable in the early stages of systemic infection (around 5 days post-inoculation [d.p.i.]) and accumulated over time as expected. For the detection of P3N-PIPO, very concentrated protein samples (near lane overloading) were required and P3N-PIPO became detectable in minute quantities only at later timepoints (around 6 d.p.i.; see also Fig3C for 21 d.p.i.), presumably after the virus had spread and accumulated within systemically infected leaves. Due to the massive differences in P3 and P3N-PIPO quantities, and rather poor detection of the latter, the frameshifting efficiency could not be determined reliably using Western analysis. Nonetheless, these experiments demonstrated that P3N-PIPO is produced only in very small amounts relative to the non-frameshift product P3.

Analysis of the ability to establish systemic infection. *Nicotiana benthamiana* plants were biolistically inoculated with WT or mutant (M1, M2, P, FSko) V5-tagged TuMV-GFP. Infection was monitored via GFP fluorescence under UV light at 5, 7 and 9 d.p.i.

Western blot and RT–PCR analysis of upper leaves of inoculated plants. Total protein extracts (21 d.p.i.) were probed with CP and V5 antibodies. Ponceau S staining of RuBisCO large subunit (RuBP-L) was used as a loading control. For RT–PCR, TuMV-specific primers were used for detection of positive-strand viral RNA.

Analysis of cell-to-cell movement. Confocal microscopy of biolistically inoculated leaves at 6 d.p.i. Scale bar = 25 μm.

### Genetic analysis indicates that −1 ribosomal frameshifting is not the primary expression mechanism for P3N-PIPO in TuMV

A highly conserved GAA_AAA_A sequence at the 5′ end of the *pipo* ORF was proposed previously to be the site of frameshifting 4 (Fig1). In TuMV, the motif is preceded by a “G”, to form a G_GAA_AAA_A sequence that might be compatible with −1 PRF to access the *pipo* ORF (Fig3A). On the other hand, conservation of the final “A” would not be relevant for −1 tandem slippage PRF but would be relevant for transcriptional slippage. To further elucidate the frameshifting mechanism, several mutants were constructed (Fig3A). Mutants M1 and M2 carry mutations 5′-adjacent to the GAA_AAA_A sequence that are expected to inhibit possible −1 PRF by preventing P-site codon:anticodon re-pairing following a −1 shift. Mutant P has mutations at the 3′ end of the GAA_AAA_A sequence that are expected to inhibit possible transcriptional slippage by reducing the length of the homopolymeric run of “A”s. Mutant FSko has mutations in the middle of the GAA_AAA_A sequence that should inhibit frameshifting by either mechanism. All the mutations listed above were introduced into the TuMV-GFP cDNA with V5-tagged P3 (denoted WT) and do not change the P3 amino acid sequence.

*Nicotiana benthamiana* plants were biolistically inoculated with WT and mutant virus clones and virus infection monitored using GFP fluorescence (Fig3B). As P3N-PIPO is required for virus movement, changes in its expression would be expected to manifest in an absence of movement or altered movement dynamics. In plants infected with WT virus, systemic infection was detected by 7 d.p.i. with GFP fluorescence detected in small clusters in the upper leaves. By 9 d.p.i., GFP fluorescence was seen over the entire leaf area of the upper leaves. Both mutants in which possible −1 PRF was inhibited, M1 and M2, behaved similarly to WT virus. In both cases, GFP fluorescence was detectable by 7 d.p.i. and reached maximum area by 9 d.p.i. In contrast, no GFP signal was detected in plants inoculated with mutant P, in which possible transcriptional slippage was inhibited, or mutant FSko, in which any type of frameshifting should be inhibited. The plants were monitored until 28 d.p.i. without any qualitative change being observable. Over two series of experiments, with 12 and 6 plants per construct, the percentages of systemic infection were as follows: WT, 75–100%; M1, 83–100%; M2, 83–100%; P, 0%; and FSko, 0%.

Upper leaves of inoculated plants were also analysed for systemic infection using Western analysis and the reverse transcription polymerase chain reaction (RT–PCR) (Fig3C). Coat protein (CP), P3 and P3N-PIPO were detected in plants inoculated with WT and mutants M1 and M2, but, as expected based on the previous results, these proteins were absent from plants inoculated with mutants P and FSko. Using RT–PCR, viral RNA was detected in plants inoculated with WT and mutants M1 and M2, but not in plants inoculated with mutants P and FSko. The cDNA fragments obtained from plants inoculated with M1 and M2 were sequenced, and no reversions were detected at the mutated sites.

Virus movement was monitored in inoculated leaves by confocal microscopy (Fig3D). For WT virus, GFP fluorescence became easily detectable from 4 to 5 d.p.i. in clusters of cells, indicating virus cell-to-cell movement. Cell clusters typically reached approximately maximum size and signal intensity by day 6. Similar cell-to-cell movement was seen with mutants M1 and M2. In contrast, with mutants P and FSko, GFP fluorescence was only detected in single cells, indicating loss of cell-to-cell movement. Thus, inhibiting the potential for transcriptional slippage at the GAA_AAA_A site resulted in a movement-deficient phenotype, probably due to no or insufficient expression of P3N-PIPO. Conversely, these experiments indicate that −1 PRF is either not used for P3N-PIPO expression in TuMV or, if it does occur at some level, it is not the primary expression mechanism.

**Figure 4:**
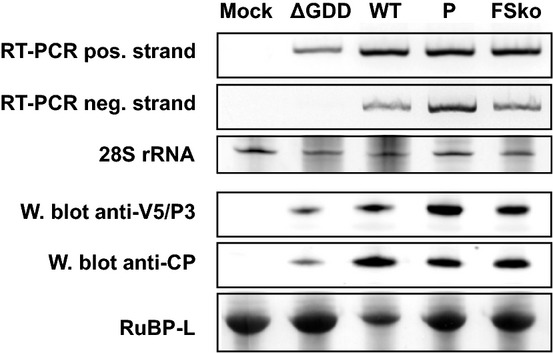
Replication of movement-deficient mutants *Nicotiana benthamiana* plants were agroinfiltrated with full-length WT and mutant viral clones. A replication-deficient mutant, ΔGDD, was used as a negative control. At 6 d.p.i., total RNA and protein were extracted from infiltrated patches. Virus replication was detected with strand-specific RT–PCR. Ethidium bromide staining of 28S rRNA was used as a control for RNA quality. Protein extracts were analysed by Western blot for the presence of CP and P3 (V5 epitope). Ponceau S staining of RuBisCO large subunit (RuBP-L) was used as a loading control.

To ensure that the movement-deficient phenotype of P and FSko was not simply an artefact of defective virus replication (with GFP fluorescence observed in single cells resulting from translation of RNA transcribed directly from the plasmid), these mutants were tested for their ability to replicate within cells. Mutants were introduced into plants by agroinfiltration (Fig4). A replication-deficient TuMV-GFP clone, ΔGDD, lacking the catalytic GDD site of the viral polymerase, was constructed and used as a control. Using strand-specific RT–PCR, the positive-strand viral RNA (derived from T-DNA transcription and/or viral replication) was, as expected, detected with all constructs: ΔGDD, WT, P and FSko. However, when strand-specific RT–PCR was used to detect the negative-strand viral RNA, which can only be produced by the viral polymerase during viral replication, cDNA fragments were detected only for WT, P and FSko, indicating that mutants P and FSko are replication competent. Viral protein accumulation was also analysed. P3 and CP were detected by Western analysis in total protein extracts from patches infiltrated with ΔGDD, WT, P and FSko. Comparison of protein levels indicated that mutants P and FSko do not accumulate as efficiently as WT virus, though this might be an artefact of the assay as WT virus would reach more cells whereas the movement-deficient mutants would only replicate and accumulate in transformed cells. As expected, much lower levels of P3 and CP were detected in patches infiltrated with the ΔGDD mutant, consistent with transcription and translation of the T-DNA encoded sequence, without viral replication. In summary, the mutants unable to move from cell to cell (P and FSko) are still able to replicate and accumulate, supporting the proposition that the movement-compromised phenotype results from the absence or insufficient expression of P3N-PIPO.

### High-throughput sequencing reveals transcriptional slippage at the GA_6_ site

**Figure 5:**
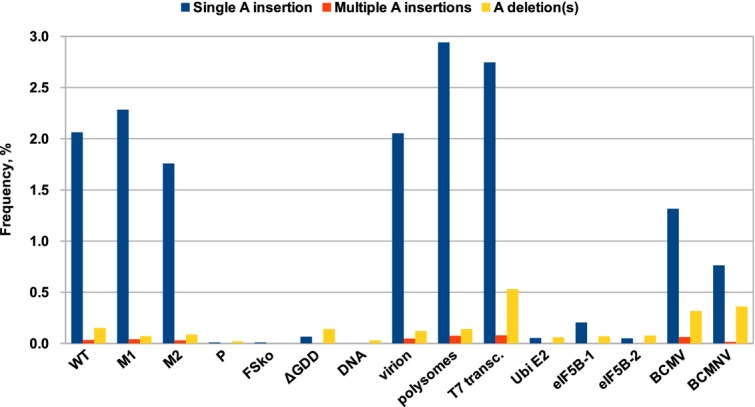
Transcriptional slippage at the GA_6_ sequence Libraries were prepared and subjected to high-throughput sequencing in order to detect low-frequency insertion/deletion events. All samples apart from Ubi E2, eIF5B-1, eIF5B-2, BCMV and BCMNV correspond to TuMV. RNA was purified from upper leaves of systemically infected plants for samples TuMV WT, TuMV M1, TuMV M2, BCMV and BCMNV. Polysomes and virions were also purified from upper leaves of TuMV-infected plants. RNA from agroinfiltrated tissue was used for the TuMV P, FSko and ΔGDD mutants. Additionally, T7 *in vitro* transcribed RNA (T7 transc.) and plasmid DNA (DNA) were analysed. Three GA_6_ sequences in host genes—Ubi E2, eIF5B-1 and eIF5B-2—were also analysed. For each sample, frequencies of transcripts with an “A” insertion at the GA_6_ sequence are shown in blue; frequencies of transcripts with two or more inserted “A” nucleotides are shown in orange; and frequencies of transcripts with one or more “A” nucleotides deleted are in yellow.

To further test whether transcriptional slippage might explain P3N-PIPO production, we performed high-throughput sequencing of the slip site region in the context of virus infection. RNA was extracted from systemically infected leaves (total RNA), as well as from virions and polysomes. Primers designed to anneal just upstream or downstream of the GA_6_ sequence were used to reverse transcribe and amplify a region surrounding the GA_6_ site, and the resulting cDNAs were subjected to high-throughput sequencing (Fig5, Appendix Table S1). Around 1.9–2.1% of reads obtained from total RNA purified from WT TuMV systemically infected leaves contained a single “A” insertion within the GA_6_ sequence (GA_6_ changed to GA_7_), thus allowing expression of P3N-PIPO at a level of ∼2%, consistent with Western blots of V5-tagged virus (Fig2). A single “A” insertion was the most abundant insertion/deletion event detected. Similar results were obtained with TuMV mutants M1 and M2, with 2.3–1.8% of reads containing a single “A” insertion. Polysomal and virion RNA (both purified from WT TuMV systemically infected leaves) were also tested. For polysomal RNA, a single “A” insertion was seen in 2.9% of reads, while, for virion-derived RNA, a single “A” insertion was seen in 2.1–2.5% of reads. Due to the movement-deficient phenotype of mutants P and FSko, RNA from agroinfiltrated patches was used to test for slippage, with insertions detected in ≤0.01% of reads. No insertions were observed when using plasmid DNA as template for library preparation, although deletions occurred at a low level (0.02–0.03%). Thus, insertions were not introduced during amplification, library preparation or sequencing.

To test whether transcriptional slippage was specific to the viral polymerase, we also tested RNAs transcribed by host cell RNA polymerase. Samples from leaf patches agroinfiltrated with the ΔGDD TuMV mutant were analysed using the same primers as above. In these samples, TuMV RNA is transcribed by *N. benthamiana* RNA polymerase II from T-DNA, although it is conceivable that some transcripts produced in infiltrated *A. tumefaciens* cells may also be present. Prior to reverse transcription, samples were excessively treated with DNase and complete elimination of contaminating DNA verified by PCR. Insertion of a single “A” at the GA_6_ site occurred at a level of 0.05–0.07% in these samples—around 33-fold lower than was seen with WT TuMV. Two host genes containing GA_6_G sequences (similar to TuMV) were also tested: *ubiquitin-conjugating enzyme E2* (*Ubi E2*) and *eukaryotic translation initiation factor 5B* (*eIF5B*). For *eIF5B*, two distinct GA_6_ sites were tested. In two of the three cases, insertion of an additional “A” at the GA_6_ site occurred at a level of 0.05–0.07% (around 33-fold below WT TuMV), while the third site was more slip-prone, with “A” insertions occurring at a level of 0.20–0.25% (around 9-fold below WT TuMV). In addition to testing specificity of higher levels of slippage to the viral polymerase, these experiments also put upper bounds on slippage introduced during reverse transcription. In contrast, the TuMV WT deletion rate (0.13–0.15%) was similar to that of the ΔGDD control (0.12–0.14%), indicating that *pipo*-site deletions are not specific to the viral polymerase.

To test whether insertions were specific to the *pipo* slip site, total RNA from systemically infected leaves and virion-derived RNA were subjected to high-throughput sequencing (ENA databank accession PRJEB9490). Similar to before, single-nucleotide insertions at the GA_6_ sequence were observed at a level of 1.9 and 2.1% for total and virion RNA, respectively. Elsewhere in the TuMV genome, an average insertion rate of 0.001% per nucleotide was seen with both samples and no other insertion “hotspots” were detected of similar magnitude to the *pipo* slip site (Appendix Fig S1).

While investigating translational frameshifting as a potential P3N-PIPO expression mechanism, we had previously performed *in vitro* translations of reporter constructs containing the *pipo* slip site and flanking sequences. We observed expression of both alternative frames with access to the *pipo* reading frame occurring at a level of 1.5–2.1% for WT sequence (Appendix Fig S2). In view of the above results, we decided to test whether slippage in the *in vitro* system might also be occurring at the level of transcription. We subjected cDNAs derived from T7-transcribed transcripts to high-throughput sequencing and found that 2.8% of transcripts had an “A” insertion in the GA_6_ sequence, while 0.5% of transcripts had deletions at the same site (Appendix Table S1). Thus, the *in vitro* frameshift products may be presumed to result from slippage by the T7 polymerase. The M1 and M2 mutations had little effect on expression of the frameshift products *in vitro* while the P mutation inhibited their production.

To confirm that transcriptional slippage was not specific to TuMV, we analysed RNA from plants infected with two other potyviruses, *Bean common mosaic virus* (BCMV; aug_GAA_AAA_Auc slip site) and *Bean common mosaic necrosis virus* (BCMNV; ucg_GAA_AAA_Auu slip site). In these species, insertion of a single “A” at the GA_6_ sequence occurred at a level of 1.3% and 0.8%, respectively, and in both an elevated level of deletions was observed in comparison with the TuMV samples. We also analysed *Plum pox virus* data available in the National Center for Biotechnology Information (NCBI) Short Read Archive (SRA) database (accession numbers ERX013141 and ERX013142; 12 libraries). The mean frequency of an “A” insertion at the GA_6_ site was 0.8%, with frequencies for individual samples ranging from 0.3 to 1.0% (Appendix Fig S3). Similarly, a low level (1–2%) of frameshift mutations at position 2,891 (which corresponds to the GA_6_ site) has been reported for *Zucchini yellow mosaic virus* 20.

Together, these results indicate that around 0.8–2% of viral transcripts produced by the potyvirus polymerase allow expression of P3N-PIPO as a consequence of an “A” insertion at the GA_6_ site. Although previous mass spectrometric analysis of cDNAs derived from RNA from TuMV-infected plants failed to detect transcripts with insertions or deletions 4, this analysis may not have been sensitive enough to identify the low level of slippage observed. While we cannot rule out −1 PRF occurring at some level in TuMV or other potyvirid species, our data suggest that transcriptional slippage alone can account for P3N-PIPO expression. An earlier hypothesis that potyviruses might use a novel +2 PRF mechanism, involving re-pairing of the P- and A-site tRNAs from GAA and AAA to A_AA and A_AN, respectively 10, now seems unnecessary and unlikely.

### Homopolymeric runs of six or more adenosines or uridines are under-represented in potyvirus genomes

If potyvirid polymerases are prone to slippage at GA_6_ sequences, selection might act against such sequences spontaneously arising at other locations (in any reading frame). In view of this, we re-ana-lysed all NCBI *Potyviridae* RefSeqs. Of 123 RefSeqs, only seven lack a GA_6_ sequence at the 5′ end of the *pipo* ORF (Appendix Dataset S1), while only twelve instances of GA_6_ sequences were found at other sites within the polyprotein ORFs (Appendix Table S2). Among potyvirid species, all three reading frames (GAA_AAA_A, G_AAA_AAA and GA_AAA_AA) are represented at the *pipo* slip site. This is consistent with a transcriptional slippage mechanism as the reading frame is relevant only for translational frameshifting. Conservation of the GA_6_ sequence suggests that transcriptional slippage is used throughout the family for P3N-PIPO expression.

To assess whether spurious GA_6_ sequences are under-represented, we generated 1,000 shuffled ORF sequences for each genus *Potyvirus* RefSeq and calculated the mean number of GA_6_ sequences in the shuffled sequences. The *bona fide pipo* slip site was excluded from the shuffling and motif counting. In 99 genus *Potyvirus* RefSeqs, we counted eight (that is, a mean of 0.081 per sequence) non-*pipo* GA_6_ sequences in the polyprotein ORF (four of which are at the proposed slip site for P1N-PISPO expression in *Sweet potato feathery mottle virus*, *Sweet potato virus 2*, *Sweet potato virus G* and *Sweet potato virus C* 21). However, in the randomized sequences, we counted a mean of 2.52 non-*pipo* GA_6_ sequences per sequence. Thus, excepting functionally utilized slip sites, GA_6_ sequences are highly under-represented in potyvirus coding sequences. Under-representation was also observed for A_7_, A_6_, U_7_ and U_6_ sequences, but not appreciably for GA_5_, A_5_ or U_5_ (Appendix Fig S4).

### Transcriptional slippage as a gene expression mechanism

Transcriptional slippage for gene expression has been reported in several bacterial genes 22,23 and as a mechanism for transposase expression in bacterial insertion sequence elements 24. In viruses of eukaryotes, transcriptional slippage has been documented in paramyxoviruses and ebolaviruses (see Introduction; 25-27,28). Transcriptional slippage has also been reported for *Hepatitis C virus* (HCV). In HCV-1, slippage on an A_10_ sequence allows expression of the F ORF, which overlaps the polyprotein ORF in the +1 reading frame 29,30. Although antibodies against F ORF peptides have been detected in chronically infected patients, it is not known whether the F ORF encodes a functional product and, furthermore, fewer than 1% of HCV isolates contain the A_10_ sequence 29. Thus, transcriptional slippage in HCV is likely accidental rather than “programmed”. Transcriptional slippage, or stuttering, also occurs in some negative-strand RNA virus taxa (including the families *Paramyxoviridae*, *Rhabdoviridae* and *Orthomyxoviridae*) for polyadenylation of the mRNA transcripts 31. Polymerase stuttering on poly(A) and poly(U) templates is also thought to maintain poly(A) tail length in picornaviruses 32. As potyvirid genomes are also polyadenylated, and the polymerase is structurally related to the poliovirus polymerase, it is possible that the potyvirid polymerase is pre-adapted to slip at homopolymeric runs of A or U.

The ability of RNA polymerases to slip on homopolymeric sequences above a certain length is believed to be linked to the length and stability of the template:nascent RNA duplex within the polymerase 33. Furthermore, some RNA polymerases appear to be inherently more slip-prone than others 33,34. The duplex length has been reported as 8–9 bp for bacterial DNA-dependent RNA polymerases and 7–8 bp for the single-subunit T7 polymerase 35-37, while RdRps of positive-sense RNA viruses in the *Picornavirus* genus constrain a duplex of 7–8 bp 38. In *Thermus thermophilus dnaX*, slippage occurs at an A_9_ sequence (T_9_ template) and is ∼50% efficient 22. The T7 polymerase slips efficiently (>50%) at U_8_ and A_8_, only modestly at U_7_, and perhaps very slightly at U_6_ 34. Consistent with this, we observed ∼2.8% T7 polymerase slippage at GA_6_ and a comparable level (0.8–2.9%) for potyviral RdRps. The role of the conserved 5′ “G” is, however, unclear. Mutation of “G” to “C” or “A” in *Soybean mosaic virus* did not prevent cell-to-cell movement 8, suggesting that the “G” is not essential for transcriptional slippage. The lack of a conserved nucleotide flanking the 3′ end of the slip site and the observation that a significant number of species have a run of seven instead of six “A”s following the “G” (Appendix Dataset S1) suggest a strand specificity in the slippage mechanism with the G:C pair likely to function during positive-strand synthesis. It is possible that the “G” plays a role in regulating the amount, direction or strand specificity of slippage. In positive-sense RNA virus transcription, unlike in negative-sense RNA virus transcription or DNA-dependent transcription, the template:nascent RNA duplex is likely to extend a considerable distance behind the RdRp footprint, possibly only being disassociated when the next RdRp passes along the template 39-41. Thus, transcriptional slippage in potyvirids may require formation of an unpaired “bulge” nucleotide. The stable G:C base-pairing may be required to limit the extent of bulging to regulate the amount of slippage or to prevent polymerase stalling or drop-off.

Transcriptional slippage poses a problem for RNA viruses because, unlike cellular organisms, the RNA is also the replicative form. Propagation of edited transcripts might lead to a homopolymeric tract of ever-increasing and variable length. The first line of defence against runaway editing may be simple selection during founder events such as transmission between hosts: infection will only be established if a functional polyprotein can be translated from the input genome(s). Ebolaviruses exist as a mixed population of genomes containing either A_7_ or A_8_ at the slippage site, with A_8_ dominating in cell culture and A_7_ dominating *in vivo* 28,42. In contrast, a specific mechanism exists in paramyxoviruses to prevent replication of edited genomes. In negative-sense RNA viruses, the genome and antigenome occur in close association with multiple copies of nucleocapsid protein. In paramyxoviruses, each nucleocapsid is associated with precisely six nucleotides of RNA, and only genomes with lengths that are a multiple of six nucleotides are efficiently replicated 43. A quite different mechanism may operate in potyvirids to prevent the replication of edited transcripts, as follows. In the potyvirus *Tobacco etch virus*, translation through a region near the 3′ end of the polyprotein ORF is required for the genome to be used efficiently as a replication template 44. It has been hypothesized that this may be due to the ribosome restructuring important RNA elements within the sequence encoding the CP, or ribosome-associated delivery of *cis*-active replication proteins. Transcripts with one or two insertions or deletions will lead to a frameshift and early termination of translating ribosomes, and therefore, these RNAs will not be replicated.

The observation of transcriptional slippage in potyviruses opens the possibility of transcriptional slippage for gene expression in other positive-sense RNA viruses, particularly those with polyadenylated genomes [whose polymerases may therefore have evolved stuttering mechanisms to maintain poly(A) tail length] and where replication is linked to translation through certain genomic regions that become inaccessible in edited transcripts. Relevant to this, many positive-sense RNA virus genomes are preferentially replicated *in cis* 45-47,48.

While this manuscript was under review, related studies were published by García and colleagues 49.

## Materials and Methods

Further details are given in Appendix Supplementary Materials and Methods.

### Viruses and plasmids

TuMV-GFP (based on isolate UK1, GenBank EF028235 50), BCMNV (PV 0413: GenBank HG792063) and BCMV (PV 0915: GenBank HG792064) were used. Mutagenesis was carried out using standard methods. For agroinfiltration, the 35S-TuMV-GFP-NosT cassette was cut from its original backbone and ligated into pGreenII.

### Inoculation and agroinfiltration

Three- to four-week-old *Nicotiana benthamiana* plants were inoculated biolistically. For cell-to-cell movement analysis, expanded younger leaves were removed from plants and biolistically inoculated. Agroinfiltration used *Agrobacterium tumefaciens* GV3101 containing the desired constructs.

### Western analysis

Leaf protein extracts were separated on 12% NuPAGE bis-tris gels, blotted to nitrocellulose membrane, probed with anti-V5 or anti-CP antibodies followed by IRdye680- or IRdye800-conjugated secondary antibodies and visualized with an Odyssey infrared scanner.

### Reverse transcription PCR

Total RNA was extracted from leaf discs 51 and RT–PCR carried out. Negative-strand-specific RT–PCR was performed as described in 52.

### Virus and polysome purification

Virions were purified from systemically infected leaves as in 53 with modifications. Polysomes were purified as in 54 with modifications 55. RNA was purified by phenol–chloroform extraction and ethanol/sodium acetate precipitation.

### High-throughput sequencing

For targeted high-throughput sequencing, PAGE-purified primers containing the sequencing adapter and target sequence were used to produce amplicons. After amplification, libraries were PAGE separated and target fragments gel purified. Libraries were sequenced using the Illumina NextSeq500 platform. Reads were preprocessed using the FASTX Toolkit (Hannon laboratory) and reads less abundant than 0.01% of the most abundant read were excluded. Insertions and deletions were quantified using custom scripts and manually verified. For whole-genome sequencing, libraries were prepared using the TruSeq Stranded mRNA Library Prep Kit (Illumina), and reads were processed using the FASTX Toolkit and mapped to the TuMV genome with BWA.

### Data accessibility

Whole-genome sequencing data are available in the ENA databank under study Accession Number PRJEB9490.

## Supporting Information

**Appendix**

**Review Process File**
