# Supplementary material for: Transcriptional slippage in the positive-sense RNA virus family Potyviridae
Source: EMBO Rep. 2015 Jun 25;16(8):995–1004. doi: 10.15252/embr.201540509 (PMC4552492; doi:10.15252/embr.201540509)
Supplement: Supplementary file 1 [file embr0016-0995-sd1.pdf]

Transcriptional slippage in the positive-sense RNA virus family *Potyviridae*

Allan Olspert, Betty Y.-W. Chung, John F. Atkins, John P. Carr, and Andrew E. Firth

Appendix

Supplementary Materials and Methods....................1
Supplementary References....................4
Figure S1....................5
Figure S2....................6
Figure S3....................7
Figure S4....................8
Table S1....................9
Table S2....................10
Dataset S1....................11

Supplementary Materials and Methods

Viruses and plasmids

The following viruses and viral constructs were used: TuMV-GFP based on isolate UK1 (GenBank accession EF028235; [1]); BCMNV isolate PV 0413 (GenBank accession HG792063), and BCMV isolate PV 0915 (GenBank accession HG792064). TuMV infections used *Nicotiana benthamiana* as the host and BCMNV/BCMV infections used *Phaseolus vulgaris* as the host. Nucleotide and amino acid coordinates for constructs and primers are relative to the RNA sequences of these accessions. Mutagenesis of constructs was carried out using overlap extension PCR with mutagenesis primers and standard cloning methods. To enable agroinfiltration, the 35S-TuMV-GFP-NosT cassette was cut out from its original backbone with *Sma*I-*Ehe*I and ligated into blunted *Kpn*I-*Sac*I sites in the vector pGreenII. A V5 tag sequence was inserted near the 5' end of the region encoding P3 to produce the protein sequence ...VG|GT**GKPIPNPLLGLDST**GTEW... (V5 tag sequence in bold, "|" indicates the P3 N-terminal processing site). Mutations made at the GAA_AAA_A conserved site are described in Fig 3A. Both the V5 tag and conserved site mutations were introduced into the vector by exchanging the *Kpn*I-*Sna*BI fragment. To generate the ΔGDD mutant, nucleotides 9014-9022 (encoding the GDD motif in the RdRp) were deleted by mutagenesis PCR and the fragment introduced back to the plasmid using *Avr*II-*Sal*I restriction sites.

Inoculation

Nicotiana benthamiana plants were grown under a 16 h photoperiod at 22 °C and 60% humidity. Three- to four-week old plants were inoculated biolistically using the PDS-1000/He system (BioRad) with 0.8-1.5 µm gold particles (AlphaAesar) coated with plasmids according to the manufacturer's instructions. For cell-to-cell movement analysis, fully expanded younger leaves roughly 3-4 cm in diameter were removed from plants, biolistically inoculated as described above, and kept in a closed container to prevent drying out of tissue prior to further analysis.

Agroinfiltration

Agrobacterium tumefaciens GV3101 containing the desired constructs was grown in LB medium at 30 °C. Bacteria were pelleted by centrifugation at 2,500 *g* at 4 °C for 15 min, resuspended in 10 mM $MgCl_2$ and pelleted again. Bacterial cells were then suspended in 0.2 mM acetosyringone in 10 mM $MgCl_2$, incubated on ice for 30 min, pelleted and resuspended in the same solution. The OD600 of bacterial suspensions was adjusted to 1.0 for agroinfiltration.

Western analysis
For protein detection, leaf disks were frozen in liquid nitrogen and homogenized. Ten volumes (v/w) of -20 °C 100% acetone was added to powdered tissue, mixed (optionally incubated at -20 °C) and centrifuged at 18,000 *g* for 15 min at 4 °C. The pellets were washed 3 times with the initial volume of -20 °C 100% acetone, centrifuging between each step, and dried under vacuum. The pellets were reconstituted in SDS loading buffer, denatured by boiling for 5 min and analysed on 12% NuPAGE bis-tris gels. Proteins were blotted to nitrocellulose membrane and blocked with 5% non-fat milk for 1 h in PBS. Membranes were then probed in PBStw with anti-V5 (LifeTechnologies) or anti-CP (DSMZ, AS-0132) antibodies for 1 h and washed three times with PBStw, followed by an incubation of 1 h with IRdye680- or IRdye800-conjugated secondary antibodies (Licor). The blots were washed twice in PBStw before visualisation with an Odyssey infrared scanner (Licor).

Reverse transcription
Leaf disks were frozen in liquid nitrogen, homogenised and total RNA was extracted as described by Oñate-Sánchez et al. [2]. Reverse transcription was carried out on 2 µg of RNA using SuperScript III (LifeTechnologies) at 50 °C according to the manufacturer's instructions. Negative-strand-specific RT was performed as described by Purcell et al. [3]. Briefly, total RNA was treated with DNase and checked for DNA contamination using PCR. Reverse transcription was done as described above using a tagged primer (ggcagtatcgtgaattcgatgcCATCAGGGTGGACAGCAACG, tag in lowercase; TuMV-GFP nt 3556-3576 in uppercase). Excess primer was removed by adding 10 U of exonuclease I (NEB) and incubated at 37 °C for 30 min followed by inactivation at 70 °C for 15 min. PCR was performed using 0.5 µl of RT reaction as template for 27 cycles with primers for the tag (ggcagtatcgtgaattcgatgc) and virus (ATGTGATTCGCCTCGGCAGT; complementary to TuMV-GFP nt 4186-4206) using Phusion DNA polymerase (NEB); cycling conditions - denaturation 20 s at 98 °C, annealing 20 s at 69 °C and extension 40 s at 72 °C. For detecting positive strand, the latter primer was used in reverse transcription, accompanied by an untagged primer (CATCAGGGTGGACAGCAACG, TuMV-GFP 3556-3576) in PCR.

Virus purification
Virions were purified as described by Baratova et al. [4] with modifications. Virions were purified from systemically infected leaves harvested 3 to 4 weeks after inoculation. Leaves were ground to powder in liquid nitrogen and 0.5 M potassium phosphate buffer, pH 7.5, containing 0.01 M DIECA, 0.005 M EDTA, and 1% (w/v) sodium sulfite (2 ml of buffer per g of leaf tissue) was added. Debris was pelleted at 8,000 *g* at 8 °C for 20 min and supernatant filtered through cloth. The supernatant was stirred for 1 h at 4 °C with 1% (v/v) Triton X100, then PEG 6000 and NaCl were added at final concentration 5% and 1.2%, respectively. The mixture was stirred at 4 °C for 2 h. The precipitate was sedimented at 8,000 *g* at 8 °C for 20 min and resuspended in 0.05 M potassium phosphate buffer, pH 7.5. Cleared supernatant was layered onto a 20% (w/v) sucrose cushion and ultracentrifuged at 150,000 *g* at 5 °C for 2.5 h. Then the pellet was resuspended in 0.05 M potassium phosphate buffer, pH 7.5 and the ultracentrifugation step with cushion repeated. For RNA extraction, virions were incubated with 1% (w/v) SDS at room temperature for 15 min, and RNA was extracted by standard phenol-chloroform extraction followed by sodium acetate precipitation.

Polysome purification
Fractions containing polysomes were purified as described by Jackson & Larkins [5] with modifications [6]. Infected tissue was homogenised in liquid nitrogen, 10 vol (v/w) of buffer (0.2 M TrisCl pH 9, 0.4 M KCl, 35 mM $MgCl_2$, 25 mM EGTA, 0.2 M sucrose, 1% v/v Triton X100, 5 mM DTT, 100 µg/µl cycloheximide) was added and the mixture was allowed to thaw slowly. The mixture was clarified by centrifugation in a Beckman JA-20 rotor at 14,300 *g* (13,500 rpm) at 4 °C

for 15 min. The supernatant was layered on top of a 4 ml cushion (40 mM TrisCl pH 9, 0.2 M KCl, 30 mM $MgCl_2$, 5 mM EGTA, 1.75 M sucrose, 5 mM DTT, 100 µg/µl cycloheximide) and ultracentrifuged in a Beckman Ti70 rotor, at 300,000 *g* (55,000 rpm) for 2 h at 4 °C. The pellet was gently rinsed and suspended (40 mM TrisCl pH 8.5, 0.2 M KCl, 30 mM $MgCl_2$, 5 mM EGTA, 100 µg/µl cycloheximide). Total RNA was extracted from suspensions using standard phenol-chloroform extraction and ethanol/sodium acetate precipitation.

High-throughput sequencing

Targeted high-throughput sequencing was performed on viral sequences containing the conserved site, or host controls with an identical GA_6 sequence. Recent *N. benthamiana* transcriptome datasets [7] were searched for transcripts containing GA_6 heptanucleotides and these were verified against other datasets (http://solgenomics.net); accession numbers below are according to Nakasugi et al. [7]. Two transcript sequences were selected: one encoding a predicted ubiquitin-conjugating enzyme E2 36 (Nbv5tr6378450), and the second encoding a predicted eukaryotic translation initiation factor 5B (Nbv5tr6430098). The latter had two separate GA_6 sites. PAGE-purified primers containing the sequencing adapter and target sequence were purchased from Integrated DNA Technologies. RT-PCR with primers containing the sequencing adapters were used to produce amplicons with the following target sequences (omitting sequencing adapters and flanking sequence included in primers): TuMV – TCCATTTTGGAAAAAAGTTA (TuMV-GFP 3824-3843, total amplified fragment 3809-3859); BCMV – AGATGGAAAAAATCTATA (BCMNV 3281-3298, total fragment 3264-3315); BCMNV – TGTGTCGGAAAAAATTTATGCAAA (BCMV 2942-2961, total fragment 2922-2978); Ubi E2 – AAAAGAAAAAGAAAAAAGAT (Nbv5tr6378450 569-588, total fragment 554-607); eIF5B-1 – CCTTTGGTAAGAAAAAAGGCAAGAA (Nbv5tr6430098 394-418, total fragment 379-433); and eIF5B-2 – TAAGATGAAGAAAAAAGGGGCTG (Nbv5tr6430098 1058-1080, total fragment 1044-1097). Due to differing target RNA abundances, reverse transcription was carried out using: 2 µg of total RNA for TuMV WT, M1 and M2; 5 µg of total RNA for mutants P and FSko, and for BCMV and BCMNV; 10 µg of total RNA for host and ΔGDD controls; 400 ng of T7 *in vitro* transcribed RNA for pT7-667; 400 ng of virion-derived RNA; and 2 µg of polysome-associated RNA. 10 µl of reverse transcription reaction was used for PCR in a final volume of 70 µl using Q5 High-Fidelity DNA Polymerase (NEB) with appropriate primers. For the DNA control 1 ng of plasmid was used as template. Libraries were amplified for 17 cycles (20 cycles for ΔGDD and host controls) of: denaturation at 98 °C for 20 s; annealing for the first five cycles at 44 °C and the rest at 60 °C for 20 s; and extension at 72 °C for 30 s. After amplification, libraries were separated on 1xTBE 10% PAGE (Life Technologies), and target fragments were cut from the gel and purified. Then the libraries were quantified fluorometrically with Qbit dsDNA HS kit (Life Technologies), normalised and sequenced using the NextSeq500 platform (Illumina). Reads were checked for quality, clipped for tailing adapter sequence and preprocessed using the FASTX Toolkit (Hannon lab): reads containing 'N's, too short reads, contaminating reads from other libraries (errors in indexing), reads with alternative transcript sequence (only detected for Ubi E2, 2-4% reads) and reads less abundant than 1/10,000 of the most abundant read (i.e. below 0.01%) were not included in the analysis. Reads were processed using custom scripts according to variability in the conserved site followed by manual verification.

For whole-genome TuMV sequencing, RNA treated with RiboZero (Epicentre) from systemically-infected leaves or untreated RNA purified from virions was used to prepare libraries with the TruSeq Stranded mRNA Library Prep Kit (Illumina) according to the manufacturer's protocol. Libraries were sequenced using the NextSeq500 platform, and reads were checked for quality and adapter sequences trimmed using the FASTX Toolkit. The resulting 25- to 76-nt reads were submitted to the ENA databank (study accession PRJEB9490) and used in the analysis. Reads were mapped to the reference virus genome with BWA [8] and positions of insertions and read coverage

were determined with custom scripts utilizing the reported CIGAR and TAG fields. Background single-nucleotide insertion rates were calculated by dividing the observed number of insertions (excluding those at the *pipo* slip site) by the product of mean read depth and genome length.

***In vitro* translation constructs**

For producing an RNA containing conserved RNA regions of the TuMV genome, a T7 promoter was inserted directly upstream of the TuMV-GFP sequence. Subsequently the following rearrangements were made: nucleotides 445 and 3347 (P1 codon 105 / P3 start) were joined with a *Kpn*I site inserted between; nucleotides 4014 and 1220 (end of PIPO / GFP sequence to extend the product) were joined with a *Nco*I site inserted between; and a TAG stop codon followed by a *Bgl*II site were inserted after nucleotide 1939 (GFP C-terminus) and joined to nucleotide 9881. This resulted in a construct, named pT7-667 (Appendix Fig S2A), which contained the following elements, in order: T7 promoter, 5'-UTR, 105 N-terminal codons of P1, P3(PIPO with a C-terminal GFP extension and stop), 3' UTR. Mutagenesis of the conserved site was carried out as described above and mutations were introduced by exchanging the *Kpn*I-*Nco*I fragment in pT7-667. For testing frameshifting *in vitro* in a virus-unrelated context, a TTG_GAA_AAA_AGT sequence, or the same fragment with mutations as described in Fig 3A, was inserted between *Xho*I-*Bgl*II sites of vector pDluc [9] to produce pDluc-12 and its derivatives. In frame controls for pT7-667 and pDluc-12 contained CTC_GAG_AAG_GT (+1 IFC) or CTC_GAG_AAG_T (+2 IFC) in place of the WT sequence TTG_GAA_AAA_AGT. All constructs were verified by sequencing.

***In vitro* translation**

Templates were linearised before transcription using *Sal*I and *Fsp*I for pT7-667 and pDluc-12 based constructs, respectively. Capped transcripts were *in vitro* transcribed using mMESSAGE mMACHINE T7 Transcription Kit (Life Technologies) according to the manufacturer's instructions. Transcripts were purified on NucAway spin columns (Life Technologies), verified for integrity by agarose gel electrophoresis and quantified. Transcripts were translated in wheat germ extract (Promega) according to the manufacturer's instructions using 125 mM potassium acetate and 400 ng of RNA in 10 µl reactions containing [^{35}S]methionine for 1.5 h at 25 °C. Reactions were terminated by addition of an equal volume of 100 µg/µl ribonuclease A and incubation at 37 °C for 20 min. Two microlitre samples were heated with the same volume of SDS-PAGE sample buffer and products analysed on 10% SDS-PAGE. The gels were dried and visualised using either autoradiography or, for quantification, phosphor storage screens and a Molecular Imager Typhoon (GE) system was used. Quantification of products was done using ImageQuant TL (GE) software.

Generation of shuffled ORF sequences

Polyprotein ORF sequences were shuffled so that the original amino acid sequence and the original total numbers of each of the 61 codons were maintained, but synonymous codons were randomly shuffled between the different sites where the corresponding amino acid is used in the original sequence. The three codons containing the *bona fide pipo* slip site were excluded from the shuffling and motif counting.

Supplementary References

1. Lellis AD, Kasschau KD, Whitham SA, Carrington JC (2002) Loss-of-susceptibility mutants of Arabidopsis thaliana reveal an essential role for eIF(iso)4E during potyvirus infection. *Curr Biol* **12:** 1046-1051
2. Oñate-Sánchez L, Vicente-Carbajosa J (2008) DNA-free RNA isolation protocols for Arabidopsis thaliana, including seeds and siliques. *BMC Res Notes* **1:** 93
3. Purcell MK, Hart AS, Kurath G, Wintona JR (2006) Strand-specific, real-time RT-PCR assays

for quantification of genomic and positive-sense RNAs of the fish rhabdovirus, Infectious hematopoietic necrosis virus. *J Virol Methods* **132:** 18-24
4. Baratova LA, Efimov AV, Dobrov EN, Fedorova NV, Hunt R, Badun GA, Ksenofontov AL, Torrance L, Järvekülg L (2001) In situ spatial organization of Potato virus A coat protein subunits as assessed by tritium bombardment. *J Virol* **75:** 9696-9702
5. Jackson AO, Larkins BA (1976) Influence of ionic strength, pH, and chelation of divalent metals on isolation of polyribosomes from tobacco leaves. *Plant Physiol* **57:** 5-10
6. Berry JO, Carr JP, Klessig DF (1988) mRNAs encoding ribulose-1,5-bisphosphate carboxylase remain bound to polysomes but are not translated in amaranth seedlings transferred to darkness. *Proc Natl Acad Sci U S A* **85:** 4190-4194
7. Nakasugi K, Crowhurst R, Bally J, Waterhouse P (2014) Combining transcriptome assemblies from multiple de novo assemblers in the allo-tetraploid plant Nicotiana benthamiana. *PLoS One* **9:** e91776
8. Li H, Durbin R (2009) Fast and accurate short read alignment with Burrows-Wheeler Transform. *Bioinformatics* **25:** 1754-1760
9. Grentzmann G, Ingram JA, Kelly PJ, Gesteland RF, Atkins JF (1998) A dual-luciferase reporter system for studying recoding signals. *RNA* **4:** 479-86
10. Firth AE (2014) Mapping overlapping functional elements embedded within the protein-coding regions of RNA viruses. *Nucleic Acids Res* **42:** 12425-12439
11. Clark CA, Davis JA, Abad JA, Cuellar WJ, Fuentes S, Kreuze JF, Gibson RW, Mukasa SB, Tugume AK, Tairo FD et al (2012) Sweetpotato viruses: 15 years of progress on understanding and managing complex diseases. *Plant Dis* **96:** 168-185

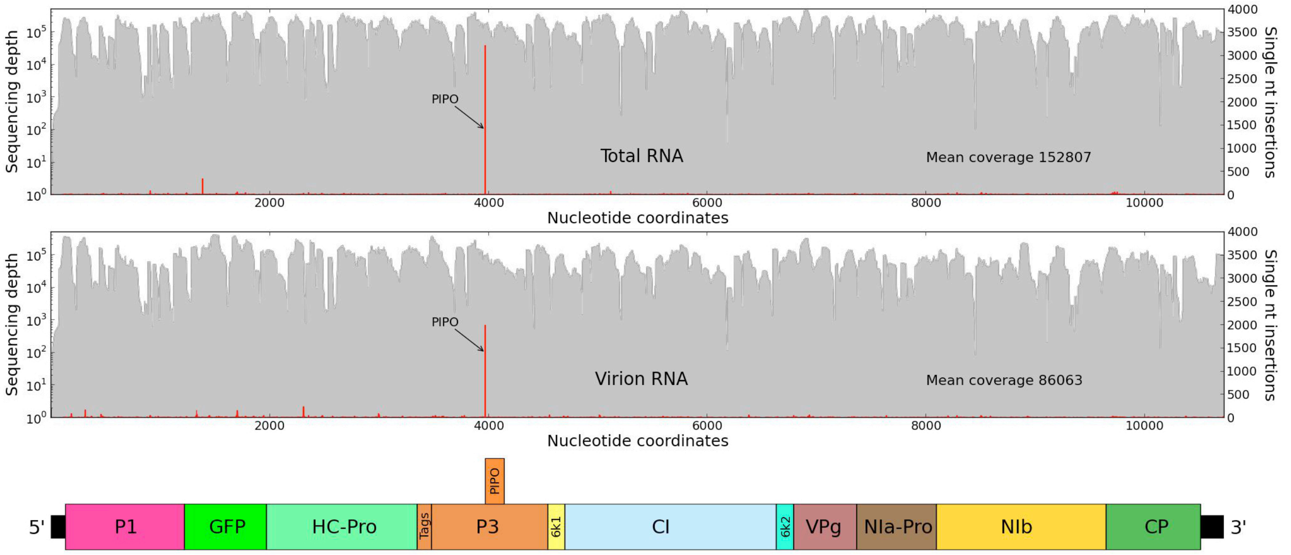

Figure S1 - Observed single nucleotide insertions in the TuMV genome. RNA from systemically infected *N. benthamiana* leaves (Total RNA) and virions (Virion RNA) was subjected to high-throughput sequencing and mapped to the TuMV genome. Sequencing depth is plotted in grey (axis on left; log scale) and observed single-nucleotide insertions are plotted in red (axis on right; linear scale). The mean total coverage is indicated for each sample. Arrows indicate the spike of insertions occurring at the GA_6 *pipo* slip site. The TuMV clone used is illustrated schematically at the bottom.

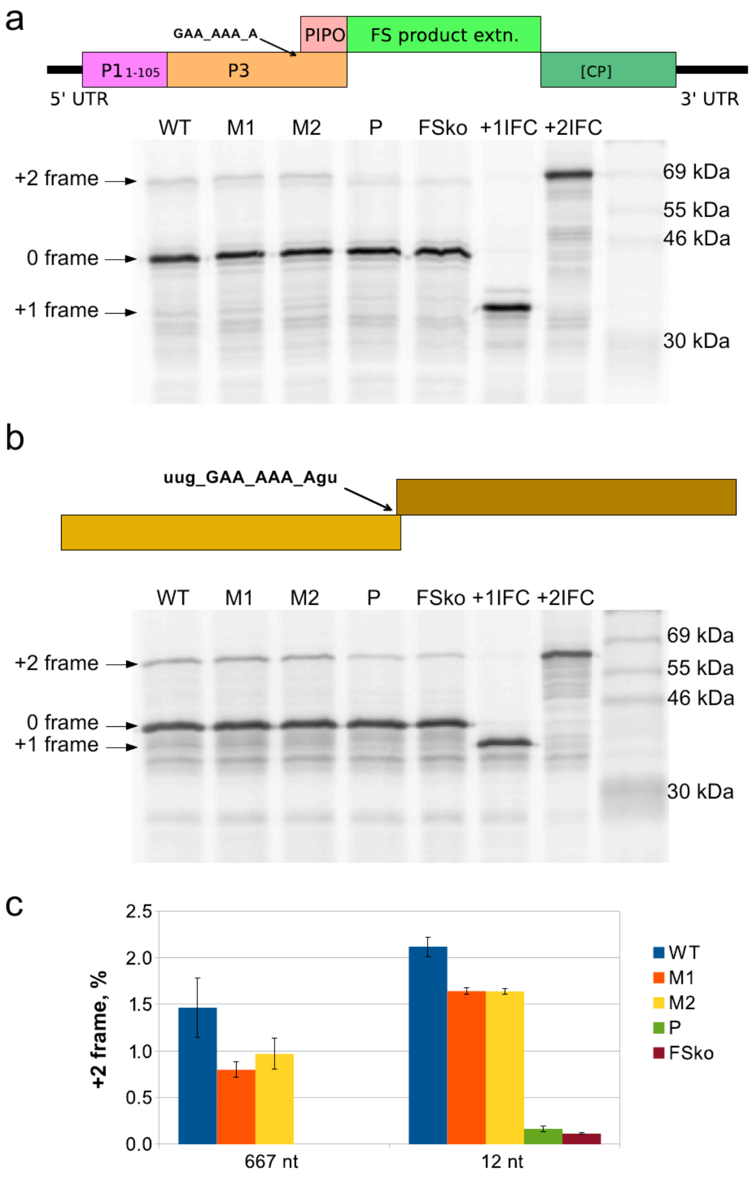

Figure S2 - Transframe expression in a T7 polymerase / *in vitro* translation system.

A *In vitro* synthesized RNAs containing 667 nt surrounding the *pipo* slip site region were translated in wheat germ extract. In case of distal stimulatory elements, TuMV-derived constructs were produced containing the full length 5' UTR and 105 codons of the 5' end of the polyprotein ORF, followed by the P3/P3N-PIPO region (667 nt) with a 3' extension of the *pipo* ORF for improved detection, followed by the 3'-terminal 165 codons of the polyprotein ORF (not translated) and the full length 3' UTR. The decision to include 105 and 165 codons from the 5'- and 3'-terminal regions of the polyprotein, respectively, was based on an analysis of conserved RNA regions in the TuMV genome [10]. A diagram of the construct is shown at top. [^{35}S]-methionine-labelled translation products from RNA with WT or mutated (M1, M2, P, FSko) slip site sequence and corresponding in-frame controls (+1IFC and +2IFC), as indicated above lanes, were analysed by SDS-PAGE. Positions of expected zero-frame and frameshift products are indicated with arrows. Molecular weight markers are shown on the right-most lane. A product corresponding to a shift into the +2 frame is observed for the WT, M1 and M2 constructs. For the same constructs, a product corresponding to a shift into the +1 frame is also observed. With mutants P and FSko, neither frameshift product is detected.

B As in (A), but using a 12-nt insert containing the conserved GAA_AAA_A sequence, mutants

thereof, or in-frame controls in an unrelated dual reporter construct. The constructs behave similarly to those with a 667-nt insert, except that the P and FSko mutations fail to completely eliminate products corresponding to a shift into the +2 frame.

C The efficiency of +2 frame expression based on the ratio of methionine-normalized densitometric volumes of +2 frame and 0 frame products. Error bars show standard deviations for 3 technical replicates. The small amount of +2 frame expression observed with the P and FSko mutants for the constructs with a 12-nt insert may result from low level translational frameshifting at the termination codon of the upstream reporter in the *in vitro* system, which would result in a product co-migrating with the product of a shift in reading frame occurring at the GA_6 sequence. Such shifting could also explain the faint band migrating slightly ahead of the +2 frame product in the constructs with the 667-nt insert. Excepting these faint bands, in view of the high-throughput sequencing analysis of T7 slippage on GA_6 (Fig 5), it would appear that the frameshift products observed *in vitro* can be explained by T7 polymerase slippage. The distal sequences included in the Fig S2A constructs were not found to stimulate translational frameshifting and are of limited relevance to an assessment of transcriptional frameshifting given the exotic polymerase (T7) and template (DNA).

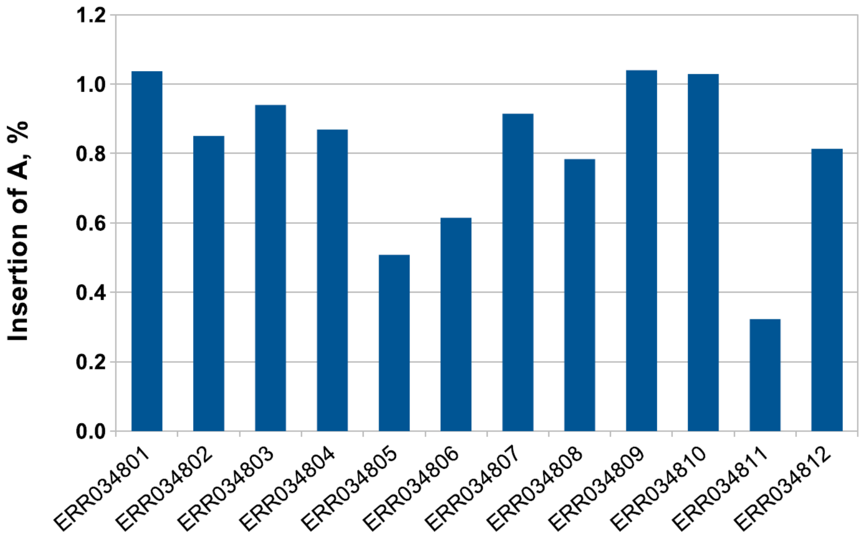

Figure S3 - Assessment of transcriptional slippage in *Plum pox virus*. NCBI SRA accession numbers ERX013141 and ERX013142 contain samples ERR034801-06 and ERR034807-12, respectively. Frequencies for reads with an additional 'A' in the conserved site (i.e. GA_6 to GA_7) are presented.

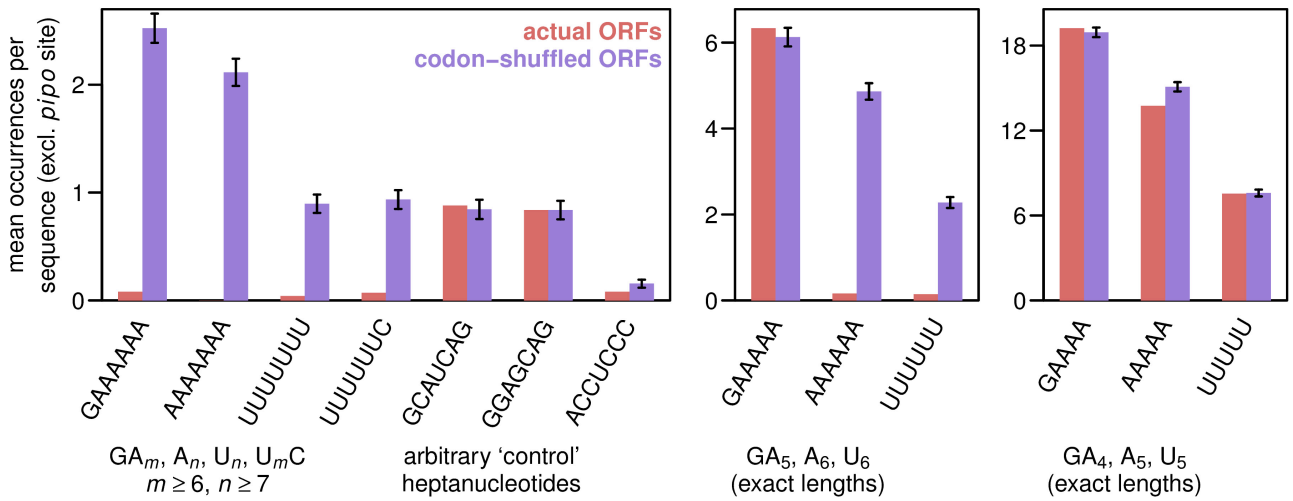

Figure S4 - Selection against A_6 and U_6 sequences in potyvirus genomes. GA_m, A_n, U_n, U_mC, GA_5, A_6, U_6, GA_4, A_5, U_5 ($m \geq 6$, $n \geq 7$) sequences were counted in the polyprotein ORF (positive-sense; any reading frame) of 99 genus *Potyvirus* NCBI RefSeqs (red bars; see Appendix Dataset S1 for accession numbers). Except for the first four motifs, only homopolymeric runs of the exact length stated were counted (e.g. the count for A_5 sequences excludes A_5 sequences that occur as part of A_6 sequences). The polyprotein ORF of each of the 99 sequences was randomly shuffled 1000 times while maintaining amino acid sequence and codon bias (see Supplementary Methods), and the frequencies of the above motifs were counted in each shuffled sequence (purple bars). Three arbitrary non homopolymeric heptanucleotides were included for comparison. The three codons containing the *pipo* slip site were excluded from these analyses. Error bars indicate standard deviations over the 1000 randomizations. Small differences between the observed and expected values (e.g. for ACCUCCC) may partly stem from dinucleotide biases which were not explicitly maintained in the randomization protocol.

Table S1. High-throughput sequencing analysis of transcriptional slippage.

Sample	# reads	Insertion of one nucleotide				Deletions and larger insertions			
		Insertion of 'A'		Other insertion		Insertion of more than one 'A'		Deletion of one or more 'A's	
		%[a]	Reads	%[a]	Reads	%[a]	Reads	%[a]	Reads
TuMV WT	5593284	2.06	115358	0.00	0	0.03	1842	0.13	7331
TuMV WT[d]	7542054	1.93	145245	0.00	0	0.03	2532	0.15	11497
TuMV M1	7802884	2.28	178130	0.00	0	0.04	3245	0.07	5827
TuMV M2	7026121	1.76	123513	0.00	0	0.03	2021	0.09	6144
TuMV P	16506489	0.01	1706	0.00	0	0.00	0	0.02	3953
TuMV FSko	17424344	0.00	0	0.00	0	0.00	0	0.00	0
TuMV ΔGDD	6302645	0.07	4133	0.00	0	0.00	0	0.14	8585
TuMV ΔGDD[d]	4865377	0.05	2489	0.00	0	0.00	0	0.12	6046
TuMV DNA	4891384	0.00	0	0.00	0	0.00	0	0.03	3475
TuMV DNA[d]	11526040	0.00	0	0.00	0	0.00	0	0.02	2115
TuMV virion	5874041	2.05	120608	0.02[b]	1016[b]	0.05	2687	0.12	6977
TuMV virion[d]	3331379	2.49	82913	0.02[b]	594[b]	0.04	1399	0.15	4995
TuMV polysomes	4779731	2.94	140552	0.00	0	0.07	3505	0.14	6675
TuMV T7 transc.	5287680	2.75	145220	0.09[c]	4628[c]	0.08	4170	0.53	28221
Host Ubi E2	3836427	0.07	2873	0.00	0	0.00	0	0.06	2179
Host Ubi E2[d]	17132433	0.05	9414	0.00	0	0.00	0	0.06	9973
Host eIF5B-1	7398964	0.25	18504	0.00	0	0.00	0	0.11	8447
Host eIF5B-1[d]	21949686	0.20	44893	0.00	0	0.00	0	0.07	16050
Host eIF5B-2	9126115	0.05	4686	0.00	0	0.00	0	0.10	8895
Host eIF5B-2[d]	20293657	0.05	9876	0.00	0	0.00	0	0.08	15490
BCMV	2126499	1.37	29234	0.00	0	0.07	1500	0.28	5977
BCMV[d]	12832374	1.32	168791	0.00	0	0.06	7984	0.32	40751
BCMNV	9783413	0.76	74500	0.00	0	0.01	1428	0.36	35418
BCMNV[d]	8341679	0.78	64828	0.00	0	0.02	1372	0.31	25809

[a] Values of '0.00' should be interpreted as '<0.01'.
[b] The 'other' sequences from the TuMV virion samples have a 'G' insertion instead of an 'A' insertion (i.e. $U_4G_3A_6G$ instead of $U_4G_2A_7G$).
[c] The 'other' sequences from the TuMV T7 transcription samples are either $U_5G_2A_6G$ (81%) or $U_4G_3A_6G$ (19%) instead of $U_4G_2A_7G$.
[d] Technical repeats.

Table S2: Non-*pipo*-associated occurrences of GA_6 sequences in NCBI *Potyviridae* RefSeqs.

genus	accession	position (nt)	sequence
Potyvirus	NC_000947.1	522	CG**GAAAAAA**CU[a]
Potyvirus	NC_009744.1	4939	UU**GAAAAAA**UA[a]
Potyvirus	NC_009745.1	600	AG**GAAAAAA**GA[a]
Potyvirus	NC_011560.1	1786	UG**GAAAAAA**CA[b]
Potyvirus	NC_001841.1	1383	UG**GAAAAAA**CU[c]
Potyvirus	NC_014742.1	1387	UG**GAAAAAA**CU[c]
Potyvirus	NC_017970.1	1231	AG**GAAAAAA**UU[c]
Potyvirus	NC_018093.1	1225	AG**GAAAAAA**UU[c]
Ipomovirus[e]	NC_010521.1	2545	AC**GAAAAAA**UC[d]
Ipomovirus[e]	NC_010521.1	7444	GG**GAAAAAA**CA[d]
Ipomovirus[e]	NC_012698.2	260	GA**GAAAAAA**GU[a]
Ipomovirus[e]	NC_014791.1	5948	GG**GAAAAAA**CA[a]

[a] GA_6 not conserved in other sequenced isolates of the same virus.
[b] Conservation of GA_6 could not be assessed due to a lack of other sequenced isolates.
[c] Potential slip site for P1N-PISPO expression in *Sweet potato feathery mottle virus*, *Sweet potato virus 2*, *Sweet potato virus G* and *Sweet potato virus C* [11].
[d] GA_6 conserved but only one other isolate available for comparison.
[e] Genus *Ipomovirus* species appear to have more spurious GA_6 sequences (four among five RefSeqs) than genus *Potyvirus* species but, while the *Ipomovirus pipo* sequence is GA_7 in the five RefSeqs (UA_7 or CA_7 in some non-RefSeq sequences), these additional sites have only 6 'A's, suggesting, potentially, that the *Ipomovirus* polymerase may require longer poly(A) sequences (i.e. A_7) for efficient slippage. Note that all 41 A_n ($n \geq 7$) sequences found in the polyprotein ORF regions of the 123 potyvirid RefSeqs occurred exclusively at the *pipo* site.

Dataset S1: Sequences at the 5' end of the *pipo* ORF in 123 NCBI *Potyviridae* RefSeqs. GA_6 sequences, as per the TuMV transcriptional slippage site, are highlighted in orange. Additional 5' 'G' and 3' 'A' residues are highlighted in pink and yellow respectively. For sequences without a canonical GA_6 site, the closest approximation is highlighted in blue. Of 99 genus *Potyvirus* RefSeqs, 88 have a GAA_AAA_A sequence and seven have a G_AAA_AAA sequence at the 5' end of the *pipo* ORF. Only four genus *Potyvirus* RefSeqs lack a GA_6 sequence at this location: one has A_AAA_AAA, two have G_AAA_AAU and one has A_AAA_AAC. It is possible that some such sequences allow low-level translational frameshifting instead of transcriptional slippage. In non-*Potyvirus* genera, 21 of 24 RefSeqs have a GA_6 sequence at the 5' end of the *pipo* ORF, with GAA_AAA_A, GA_AAA_AA and G_AAA_AAA all represented in different species. Some species have longer 'A' tracts, e.g. GA_7 in all current *Ipomovirus*, *Macluravirus* and *Poacevirus* RefSeqs. The other three non-*Potyvirus* RefSeqs have GCA_AAA_AA, G_CAA_AAA_A and A_AAA_AAA_A sequences. The A_8 sequence is likely to be consistent with transcriptional slippage while the GCA_6 sequence (which occurs in all sequenced isolates of the *Bymovirus Barley mild mosaic virus* and the *Tritimovirus Yellow oat-grass mosaic virus*, but in a different reading frame for the two species) is presumably a viable variant of the slippage site.

Potyvirus

NC_004013	UCA	GCA	CGG	**GAA**	**AAA**	**A**UC	UAU	GUC	*Cowpea aphid-borne mosaic virus*
NC_011560	AUC	GAG	CGG	**GAA**	**AAA**	**A**UU	UUU	GUU	*Zantedeschia mild mosaic virus*
NC_014064	GAA	AUG	CGG	**GAA**	**AAA**	**A**UA	UAU	GCA	*Freesia mosaic virus*
NC_016159	GAC	ACA	UGG	**GAA**	**AAA**	**A**UC	UAC	GCG	*Keunjorong mosaic virus*
NC_019415	AAU	GAA	AGG	**GAA**	**AAA**	**A**UU	UAC	UUA	*Blue squill virus A*
NC_000947	CAA	CUC	AUG	**GAA**	**AAA**	**A**AU	UAU	CUA	*Japanese yam mosaic virus*
NC_001616	CAG	AUU	AUG	**GAA**	**AAA**	**A**AU	UAU	CUA	*Potato virus Y*
NC_003398	ACC	CUA	AUG	**GAA**	**AAA**	**A**AU	UAU	GUA	*Sugarcane mosaic virus*
NC_003399	GAA	GUG	AUG	**GAA**	**AAA**	**A**AU	UAU	CUG	*Scallion mosaic virus*
NC_005288	GAG	CUU	AUG	**GAA**	**AAA**	**A**AU	UAU	CUG	*Lily mottle virus*
NC_011541	GAA	GUA	AUG	**GAA**	**AAA**	**A**AU	UAU	CUG	*Narcissus yellow stripe virus*
NC_014325	AAA	CUU	AUG	**GAA**	**AAA**	**A**AU	UAU	CUC	*Bidens mottle virus*
NC_023014	CAC	UUA	AUG	**GAA**	**AAA**	**A**AU	UAU	CUA	*Bidens mosaic virus*
NC_023628	GAG	GUG	AUG	**GAA**	**AAA**	**A**AU	UAC	UUG	*Narcissus late season yellows virus*
NC_020105	UAU	ACU	AUG	**GAA**	**AAA**	**A**AC	UAU	CAA	*Brugmansia mosaic virus*
NC_003536	GUA	CUC	AUG	**GAA**	**AAA**	**A**UC	UGG	GCA	*Clover yellow vein virus*
NC_005778	CAC	AUC	AUG	**GAA**	**AAA**	**A**UC	UAC	AAG	*Chilli veinal mottle virus*
NC_007728	GUA	GAA	AUG	**GAA**	**AAA**	**A**UC	UAU	GUA	*East Asian passiflora virus*
NC_009742	UCA	CAG	AUG	**GAA**	**AAA**	**A**UC	UAU	GUC	*Telosma mosaic virus*
NC_009744	CAU	UUA	AUG	**GAA**	**AAA**	**A**UC	UAC	AAG	*Wild tomato mosaic virus*
NC_014742	GAA	CUC	AUG	**GAA**	**AAA**	**A**UC	UGG	GAC	*Sweet potato virus C*
NC_014905	GAA	ACA	AUG	**GAA**	**AAA**	**A**UC	UAU	CAG	*Apium virus Y*
NC_018833	GCA	AUG	AUG	**GAA**	**AAA**	**A**UC	UAC	AUC	*Iranian johnsongrass mosaic virus*
NC_001841	GAG	CUU	AUG	**GAA**	**AAA**	**A**UU	UGG	GAU	*Sweet potato feathery mottle virus*
NC_003397	GUU	GAA	AUG	**GAA**	**AAA**	**A**UU	UAU	AUA	*Bean common mosaic virus*
NC_015393	GAA	ACC	AUG	**GAA**	**AAA**	**A**UU	UAU	CAA	*Celery mosaic virus*
NC_017970	GAG	AUG	AUG	**GAA**	**AAA**	**A**UU	UGG	GAU	*Sweet potato virus 2*
NC_009741	GAA	GCA	AUG	**GAA**	**AAA**	**A**UG	UAC	CAG	*Basella rugose mosaic virus*
NC_001768	GAG	AUA	AUG	**GAA**	**AAA**	**A**CC	UAU	CUC	*Tobacco vein mottling virus*
NC_003492	AUU	UUA	AUG	**GAA**	**AAA**	**A**CC	UUA	AUG	*Bean yellow mosaic virus*
NC_004035	UCA	UUG	AUG	**GAA**	**AAA**	**A**GU	UAC	GUC	*Sorghum mosaic virus*
NC_005028	UAU	UGG	AUG	**GAA**	**AAA**	**A**GU	UAU	CUC	*Papaya leaf-distortion mosaic virus*
NC_008393	CGG	AUU	AUG	**GAA**	**AAA**	**A**GU	UAU	CUC	*Pepper severe mosaic virus*
NC_010735	AAA	AUC	AUG	**GAA**	**AAA**	**A**GU	UAU	CUA	*Verbena virus Y*
NC_014038	CAC	CUC	AUG	**GAA**	**AAA**	**A**GU	UAU	CUA	*Sunflower chlorotic mottle virus*
NC_017824	AAG	GUG	AUG	**GAA**	**AAA**	**A**GU	UAU	CUC	*Tomato necrotic stunt virus*

NC_020072	GCA	CUC	AUG	**GAA**	**AAA**	**A**GC	UAU	GCA	*Colombian datura virus*
NC_001517	UGG	ACC	UUG	**GAA**	**AAA**	**A**AU	UAU	CAA	*Pepper mottle virus*
NC_004573	GAG	UCA	UUG	**GAA**	**AAA**	**A**AU	UAC	AGC	*Peru tomato mosaic virus*
NC_001555	GAA	AUG	UUG	**GAA**	**AAA**	**A**AC	UAU	GUA	*Tobacco etch virus*
NC_010954	GUU	GAA	UUG	**GAA**	**AAA**	**A**AC	UAU	GUG	*Fritillary virus Y*
NC_017967	AGC	UAU	UUG	**GAA**	**AAA**	**A**AC	UAC	ACA	*Hippeastrum mosaic virus*
NC_025250	GAA	CUC	UUG	**GAA**	**AAA**	**A**AC	UAC	AAG	*Vanilla distortion mosaic virus*
NC_002509	UCC	AUU	UUG	**GAA**	**AAA**	**A**GU	UAU	CUA	*Turnip mosaic virus*
NC_001785	GAA	GCU	GUG	**GAA**	**AAA**	**A**CC	UAC	GCC	*Papaya ringspot virus*
NC_023175	GUU	GCU	GUG	**GAA**	**AAA**	**A**CC	UAC	GCC	*Zucchini tigre mosaic virus*
NC_016044	CAU	CUU	GUG	**GAA**	**AAA**	**A**UU	UAU	CAA	*Chilli ringspot virus*
NC_018093	GAA	CUC	CUG	**GAA**	**AAA**	**A**UU	UGG	GAA	*Sweet potato virus G*
NC_004047	CAU	GUG	UCG	**GAA**	**AAA**	**A**UU	UAU	GCA	*Bean common mosaic necrosis virus*
NC_005304	UCU	CUC	ACG	**GAA**	**AAA**	**A**UG	UAU	GUG	*Beet mosaic virus*
NC_011918	AGU	GUG	CUU	**GAA**	**AAA**	**A**UC	UGC	AGG	*Pepper veinal mottle virus*
NC_016441	AUU	GAA	CUU	**GAA**	**AAA**	**A**UG	UAU	AUA	*Yam bean mosaic virus*
NC_007913	GAA	AUG	CUU	**GAA**	**AAA**	**A**AU	UAU	CUC	*Konjac mosaic virus*
NC_014327	CGA	AUA	CUU	**GAA**	**AAA**	**A**AC	UAU	CUC	*Pepper yellow mosaic virus*
NC_004010	CAA	GUC	CUU	**GAA**	**AAA**	**A**AC	UAC	AUG	*Potato virus V*
NC_004426	GAA	ACU	CUU	**GAA**	**AAA**	**A**GU	UAC	ACA	*Wild potato mosaic virus*
NC_007180	GAA	AUU	CUU	**GAA**	**AAA**	**A**GC	UAC	AUG	*Thunberg fritillary virus*
NC_007147	UCA	CUU	GUU	**GAA**	**AAA**	**A**UC	UAC	AUC	*Pennisetum mosaic virus*
NC_009995	GUU	GCA	GUU	**GAA**	**AAA**	**A**UC	UAC	ACG	*Moroccan watermelon mosaic virus*
NC_004752	CAU	CAU	GUU	**GAA**	**AAA**	**A**AC	UAU	GUG	*Yam mosaic virus*
NC_010736	GCA	GCG	GUU	**GAA**	**AAA**	**A**CU	UAC	GUC	*Algerian watermelon mosaic virus*
NC_009994	GCA	CUC	AUU	**GAA**	**AAA**	**A**UC	UAU	CAA	*Tobacco vein banding mosaic virus*
NC_001671	GCU	CUA	AUU	**GAA**	**AAA**	**A**AC	UAU	CAA	*Pea seed-borne mosaic virus*
NC_019409	GAA	AUC	UUU	**GAA**	**AAA**	**A**AU	UAU	UGC	*Ornithogalum mosaic virus*
NC_018176	AGU	AUU	AUU	**GAA**	**AAA**	**A**GU	UAC	GCA	*Arracacha mottle virus*
NC_002634	AUG	GCA	UAU	**GAA**	**AAA**	**A**UC	UAC	UCA	*Soybean mosaic virus*
NC_007216	AUA	GUG	UAU	**GAA**	**AAA**	**A**UC	UAU	GUA	*Wisteria vein mosaic virus*
NC_004011	GAA	AUC	UAU	**GAA**	**AAA**	**A**AU	UAU	CAG	*Leek yellow stripe virus*
NC_006262	GUA	ACG	CAU	**GAA**	**AAA**	**A**UC	UAU	GUG	*Watermelon mosaic virus*
NC_014252	GAA	ACC	ACU	**GAA**	**AAA**	**A**UC	UAC	AUA	*Panax virus Y*
NC_019412	UCA	CUA	ACU	**GAA**	**AAA**	**A**UU	UAU	GUA	*Yam mild mosaic virus*
NC_002600	GAA	AUG	CGU	**GAA**	**AAA**	**A**UU	AUU	GGA	*Peanut mottle virus*
NC_003537	AGU	GAG	CGU	**GAA**	**AAA**	**A**UU	UUU	GUA	*Dasheen mosaic virus*
NC_021196	AUA	GUG	UGU	**GAA**	**AAA**	**A**UU	UAU	GUG	*Calla lily latent virus*
NC_003224	GAU	AUG	AGA	**GAA**	**AAA**	**A**UG	UAU	GCA	*Zucchini yellow mosaic virus*
NC_014790	AGC	GCA	AGA	**GAA**	**AAA**	**A**UU	UAU	GCA	*Passion fruit woodiness virus*
NC_015394	AUG	GAG	AGA	**GAA**	**AAA**	**A**UC	UUU	CUU	*Hardenbergia mosaic virus*
NC_004039	GAA	CUU	CUA	**GAA**	**AAA**	**A**CU	UAC	UUG	*Potato virus A*
NC_021786	CGA	GUG	CUA	**GAA**	**AAA**	**A**UU	UAC	ACA	*Habenaria mosaic virus*
NC_021065	AGU	UUA	UUA	**GAA**	**AAA**	**A**AC	UGG	GAA	*Sunflower mild mosaic virus*
NC_001445	CAC	UUG	GUA	**GAA**	**AAA**	**A**GU	UAU	CUC	*Plum pox virus*
NC_003605	GAA	CUC	AUA	**GAA**	**AAA**	**A**GU	UAU	CUC	*Lettuce mosaic virus*
NC_009745	GCA	UUC	UCA	**GAA**	**AAA**	**A**AA	UAU	CAA	*Banana bract mosaic virus*
NC_003377	GCC	AUC	AUC	**GAA**	**AAA**	**A**CC	UAU	GUA	*Maize dwarf mosaic virus*
NC_008028	AUA	GCU	AUC	**GAA**	**AAA**	**A**AC	UAU	CAA	*Daphne mosaic virus*
NC_025254	GCA	AUG	CUC	**GAA**	**AAA**	**A**GC	UAU	CUG	*Carrot thin leaf virus*
NC_018872	GAA	AUU	UGC	**GAA**	**AAA**	**A**AU	UAC	AUC	*Pokeweed mosaic virus*
NC_003742	GAC	AGA	AUU	GA**G**	**AAA**	**AAA**	UAC	AUC	*Cocksfoot streak virus*
NC_013261	GAA	AUG	AUC	GA**G**	**AAA**	**AAA**	UAC	GCA	*Canna yellow streak virus*
NC_020896	GAG	ACA	CUC	GA**G**	**AAA**	**AAA**	UAU	GCC	*Sweet potato latent virus*
NC_025821	GGU	UUU	ACU	GA**G**	**AAA**	**AAA**	UAU	CUG	*Asparagus virus 1*
NC_005029	GAA	ACA	AA**G**	**AAA**	**AAA**	UGU	UAC	GAC		*Onion yellow dwarf virus*
NC_007433	GAG	AUG	AA**G**	**AAA**	**AAA**	UGU	UUC	GAC		*Shallot yellow stripe virus*

	NC_017977	AA**G AAA AAA** GAA GAU UUG UAU GUG	*Vallota speciosa virus*
	NC_021197	**GAA AAA A**GA GAG GAA UGG UAC AGA	*Donkey orchid virus A*
*	NC_008824	AUA AAA AAA GAA GAA UUG CUA GUA	*Narcissus degeneration virus*
	NC_003606	GAG AUA AUU GAG AAA AAU UAC GCA	*Johnsongrass mosaic virus*
	NC_014536	GAG UCA AUG GAG AAA AAU UAU CAA	*Brugmansia suaveolens mottle virus*

* AUA AAA AAA GAA is altered to AUC AAA AAA GAA in the other available isolate of *Narcissus degeneration virus*.

†	NC_014898	AUG UUA AAA AAC ACA UCA AGC GUG	*Lupine mosaic virus*

† The A AAA AA sequence in NC_014898 aligns ~8 codons upstream of the canonical GAA AAA A site.

Brambyvirus

NC_008558	CAC GUU GA**G AAA AAA** GAA CAG CUC	*Blackberry virus Y*

Bymovirus

	NC_002350	AUC AGC G**GA AAA AA**U CGC GUA GAA	*Wheat yellow mosaic virus*
	NC_002990	CUG AGU G**GA AAA AA**U CGC GUA GAA	*Barley yellow mosaic virus*
	NC_004016	CUA AGC G**GA AAA AA**U CGC AUC GAA	*Oat mosaic virus*
*	NC_003483	AUC GCA GCA AAA AAC CGC AUC GAA	*Barley mild mosaic virus*

* GCA AAA AAY (Y = C or U) is conserved in all isolates of *Barley mild mosaic virus* (9 sequences).

Ipomovirus

NC_010521	CGU GUC GUG **GAA AAA A**AG ACU CAU	*Squash vein yellowing virus*
NC_003797	AGA AUC CUU **GAA AAA A**AC AUU GGA	*Sweet potato mild mottle virus*
NC_006941	CGA AUC AUU GA**G AAA AAA** ACA AAA	*Cucumber vein yellowing virus*
NC_012698	AGG AUC AUU GA**G AAA AAA** AGA GAG	*Cassava brown streak virus*
NC_014791	AGA AUA AUU GA**G AAA AAA** ACA CAG	*Ugandan cassava brown streak virus*

Macluravirus

NC_018455	CUC AUC AAG **GAA AAA A**AC UAC AUG	*Chinese yam necrotic mosaic virus*

Poacevirus

NC_012799	UUC AGU ACA AG**G AAA AAA** ACA UUA	*Triticum mosaic virus*
NC_014037	UUU GGU GAG **GAA AAA A**AC ACU AUC	*Sugarcane streak mosaic virus*
NC_018572	UUU UAC GA**G AAA AAA** AGU ACU AUA	*Caladenia virus A*

Unclassified

NC_019031	GUU GUA UCA AAA AAA AUG GAU UUC	*Rose yellow mosaic virus*

Rymovirus

NC_001814	UGG CGA AA**G AAA AAA** GAG CAA UAC	*Ryegrass mosaic virus*
NC_005903	AUG CGA CG**G AAA AAA** GAA CUG UAU	*Agropyron mosaic virus*
NC_005904	AUG CGG AA**G AAA AAA** GAA CAG UAU	*Hordeum mosaic virus*

Tritimovirus

	NC_003501	GAA G**GA AAA AA**A UCU AUA CUC GAA	*Brome streak mosaic virus*
	NC_022745	GAU G**GA AAA AA**A AAA AUG CUC GUA	*Tall oatgrass mosaic virus*
	NC_001886	GGA GGA AAG **GAA AAA A**UG CUC GUC	*Wheat streak mosaic virus*
	NC_005136	GGA GGC AAG **GAA AAA A**UG CUC GUC	*Oat necrotic mottle virus*
	NC_009805	GAU GGU AA**G AAA AAA** AUC UUG GAA	*Wheat eqlid mosaic virus*
*	NC_024471	GAC ACA AAG CAA AAA AUG CUC GUC	*Yellow oat-grass mosaic virus*

* G CAA AAA AU is conserved in all isolates of *Yellow oat-grass mosaic virus* (2 sequences).
