# Supplementary material for: Transcriptional slippage in the positive-sense RNA virus family Potyviridae
Source: EMBO Rep. 2015 Jun 25;16(8):995–1004. doi: 10.15252/embr.201540509 (PMC4552492; doi:10.15252/embr.201540509)
Supplement: Supplementary file 2 [file embr0016-0995-sd2.pdf]

EMBO
reports

Manuscript EMBOR-2015-40509

Transcriptional slippage in the positive-sense RNA virus family Potyviridae

Allan Olspert, Betty Y.-W. Chung, John F. Atkins, John P. Carr and Andrew E. Firth

Corresponding author: Andrew E. Firth, University of Cambridge

Review timeline:

Submission date:	08 April 2015
Editorial Decision:	28 April 2015
Revision received:	29 May 2015
Editorial Decision:	03 June 2015
Revision received:	05 June 2015
Accepted:	08 June 2015

Transaction Report:

(Note: With the exception of the correction of typographical or spelling errors that could be a source of ambiguity, letters and reports are not edited. The original formatting of letters and referee reports may not be reflected in this compilation.)

Editor: Nonia Pariente

1st Editorial Decision 28 April 2015

Thank you for your patience during the peer-review of your study at EMBO reports. As you will see, although the referees raise a few issues mainly regarding controls or minor revisions, they all find the topic of interest and suitable for publication in EMBO reports.

As the requested changes are minor and all seem pertinent, I think all should be addressed. Given the sensitivity of the timing in this case, it would of course make sense to try to speed up revision as much as possible. In this regard, I am unsure of you have data at hand that could be analyzed to address referee 3's point 2. If not, addressing this issue would not be a precondition for acceptance.

If the referee concerns can be adequately addressed, we would be happy to accept your manuscript for publication. Please note that it is EMBO reports policy to undergo one round of revision only and thus, acceptance of your study will depend on the completeness of your revision, which may be sent to the referees. In this case, I would like to set 6 weeks as a revision due date. Do you think this would suffice? If not, please get in touch to discuss a timeline.

Your study will be published in report format. This means that you will have to assemble the existing data into a maximum of 5 figures, which seems relatively straighforward for your study. In addition, reports include a merged "Results & Discussion" section and abridged Materials and Methods (basic Materials and Methods required for understanding the experiments performed must remain in the main text, but additional detailed information necessary to reproduce them may be included as Supplementary Material). Revised manuscript length must be a maximum of 35,000 characters (including spaces).

© European Molecular Biology Organization

Please note that it is a precondition for publication in EMBO reports that authors agree to make all data that cannot be published in the journal itself freely available, where possible in an appropriate public database. This should be specified in the main text in the first instance where the data are mentioned, with the relevant accession code (which can also be included in the Methods section under a "Data accessibility" subheading). In the case of nucleotide sequence datasets, they should be submitted to an International Nucleotide Sequence Database Collaboration member: GenBank (http://www.ncbi.nlm.nih.gov/genbank/), EMBL Nucleotide Sequence Database (http://www.ebi.ac.uk/embl/) or DDBJ (http://www.ddbj.nig.ac.jp/).

I look forward to seeing a revised form of your manuscript when it is ready. In the meantime, please contact me if I can be of any assistance.

REFEREE REPORTS:

Referee #1:

This manuscript describes the discovery of a polymerase slippage mechanism for mediating expression of an RNA plant virus gene. In the realm of plant virology, this represents a new phenomenon and adds a novel process to the known collection of gene expression strategies. The authors have done a very thorough job and have provided substantial computational and biological evidence to support the conclusions drawn. Overall, it is a very solid and important piece of work that will be appreciated by a wide audience. A few minor suggestions for improvement are provided below.

1. Add the calculated molecular masses to the right of P3 and P3NPIPO in Figure 1A

2. Line 126-127 - need to state that these results are "(data not shown)"

3. Line 229 - it would also be important to test to see if the reverse transcriptase used in cloning the viral RNA is able to slip at this site and contribute to the indels observed

4. Would the P3NPIPO encoding genomes (i.e. with the extra A) that were detected in virus particles be viable? That is, how essential is the full-length P3 protein for infections? The ~2% trade-off is probably negligible when weighted against the need to express the transframe protein. However, the low level of slippage observed could be, in part, the consequence of selection to minimize the production of such defective genomes. This may be worth mentioning.

5. Could minus strand be analyzed to help determine if the slippage occurs during plus or minus strand synthesis?

6. This reviewer has also read the very recently published (April 15th) short communication (i.e. Letter to the Editor) on this subject by the Garcia lab in Journal of Virology. This article and the current manuscript both provide data supporting a slippage mechanism in potyviruses. However, the current manuscript provides a much more comprehensive study that, importantly, includes in planta experiments that support the biological relevance of this phenomenon. Consequently, this work will be widely recognized as an important contribution in this area of research.

7. The discussion, though interesting, seems a bit drawn out - and thus would benefit from a bit of judicious trimming.

Referee #2:

The Potyviridae family of viruses is comprised of positive-sense, single-stranded RNA viruses that are infectious to many plant species and are responsible for more than half of viral crop damage globally. Recently, a conserved overlapping gene in the -1/+2 frame designated as pipo has been identified in the P3-encoding region of the viral polyprotein coding sequence. PIPO is expressed as

a fusion protein with the N-terminus of P3, and is essential for virus cell-to-cell transmission. While the mechanism underlying the expression of PIPO was previously suggested to be via a programmed frameshifting mechanism, the current work demonstrates that PIPO expression occurs via transcriptional slippage by the viral RNA polymerase, which generates a minor subset of viral transcripts that contain an additional adenosine insertion within a conserved GAAAAAA sequence. Mutations that inhibit transcriptional slippage or prevent expression of PIPO prevented viral spread to the upper leaves following biolistic inoculation of Nicotiana benthamiana plants. High throughput sequencing indicated a small enrichment of a single adenosine insertion event in WT, M1 and M2-infected samples compared to the P and FSko-infected samples, and similarly in purified virions and polysome fractions, in support of transcriptional slippage as the mechanism for PIPO expression. Overall, the finding is significant and resolves the mechanism of pipo expression in this virus. The results are convincing and the conclusions are supported by the results. I only have minor comments that improve or clarify their conclusions.
Comments:
1. On Page 5, lines 127-128, a statement was made that "The signal strengths and expression did not change further during later stages of infection (7 to 21 d.p.i)." A western blot showing a time point late in infection (ie. 21 d.p.i) should be included in Fig. 2 to support this.
2. In Fig. 4b, it would be informative to also include the western blot for anti-V5/P3N-PIPO.
4. Do M1 and M2 mutations affect viral titer/infectivity, which may suggest a minor contribution of -1 PRF for PIPO expression?
5. From Fig. 5, it is difficult to quantitatively assess if the mutants have defects in replication based on RT-PCR. Northern blot or real-time RT-PCR should be performed and a normalizing control (other than EtBr staining of rRNA) should be used.

Referee #3:

SUMMARY
In this manuscript Olspert et al. analyse the molecular mechanism that leads to the expression of the P3N-PIPO gene product in the Potyviridae. The authors attempt to unravel whether a translational or a transcriptional frame shifting is responsible for the production of this alternative protein. The authors engineer viruses expressing GFP and encoding a N-terminal-V5-tagged P3 and P3N-PIPO proteins and first demonstrate that systemic leaves of infected plants express both proteins, the latter accounting for about 2% of total. Then the authors generate point mutant viruses, which would impede ribosomal frame shifting, transcriptional frame shifting or both and analyse the expression of GFP in systemic leaves of infected plants. Results show that mutants affecting transcriptional frame shifting are affected in GFP expression but mutants affecting ribosomal frame shifting are not. As a control, the authors show that replication and expression of all these mutant viruses in agro-infected cells is normal. Finally, the authors carry out deep sequencing of the frame-shifting region of the genome from virus infected cellular RNA and virion RNA and show that only mutations affecting transcriptional frame shifting abolish the insertion of a single A residue at the frame shifting point.

GENERAL COMMENTS
The manuscript is well written (but see below) and the figures are clear. The experiments are well performed and controlled. The results clearly support the conclusions put forward. I include below some comments and suggestions for improvement.

SPECIFIC POINTS
1. Results. Lines 131-134.
The experiments do not tell about the requirement of P3N-PIPO.
If the levels of expression of P3N-PIPO were so small that efficiency of frame shifting cannot be determined, a change in the proportion of P3/P3N-PIPO would not be detectable either.

2. Results. Section on deep sequencing.
It would have been interesting to perform whole-genome deep sequencing of virus infected cells and virions to test whether other positions in the viral RNA show insertions or deletions in a proportion similar to that detected in the GA6 site.

3. Results. Lines 279-322.
The description of results in this section is rather cumbersome. It could be simplified to make it shorter and more amenable to the readers.

4. Discussion.
Discussion is unnecessarily long and at some points reiterative. It could be pruned and simplified.

5. Discussion. Lines 401-403
The role of the 5'-G is discussed but it could have been tested experimentally.

MINOR POINT
1. Introduction. Line 73. DownStream.

1st Revision - authors' response 29 May 2015

Authors' point-by-point response

We thank all three reviewers for their positive assessment and their helpful and insightful comments, which we have addressed in detail below.

Editor's comments:

Your study will be published in report format. This means that you will have to assemble the existing data into a maximum of 5 figures, which seems relatively straighforward for your study. In addition, reports include a merged "Results & Discussion" section and abridged Materials and Methods (basic Materials and Methods required for understanding the experiments performed must remain in the main text, but additional detailed information necessary to reproduce them may be included as Supplementary Material). Revised manuscript length must be a maximum of 35,000 characters (including spaces).

We have shortened and streamlined the Discussion, moved Methods and Materials details, Figure 7, Table 1, and part of the bioinformatic analysis to the Appendix, and removed some overlap between the Introduction and Discussion. Figures 3 and 4 have been merged. We have shortened the manuscript as much as possible, and have managed to reduce the character count from 51517 to 35273, even with the addition of the new data.

Referee #1:

This manuscript describes the discovery of a polymerase slippage mechanism for mediating expression of an RNA plant virus gene. In the realm of plant virology, this represents a new phenomenon and adds a novel process to the known collection of gene expression strategies. The authors have done a very thorough job and have provided substantial computational and biological evidence to support the conclusions drawn. Overall, it is a very solid and important piece of work that will be appreciated by a wide audience. A few minor suggestions for improvement are provided below.

1. Add the calculated molecular masses to the right of P3 and P3NPIPO in Figure 1A

Edit applied.

2. Line 126-127 - need to state that these results are "(data not shown)"

We have now added a reference here to Fig 3C (previously Fig 4B) which now shows both P3 and P3N-PIPO (anti-V5 WB) at 21 d.p.i.. The text now reads "... P3N-PIPO became detectable in minute quantities only at later timepoints (around 6 d.p.i.; see also Fig 3C for 21 d.p.i.)" (lines 122-124) (line numbering as per revised version).

© European Molecular Biology Organization

3. Line 229 - it would also be important to test to see if the reverse transcriptase used in cloning the viral RNA is able to slip at this site and contribute to the indels observed

We agree entirely. This is dealt with in the lines preceding the statement "As well as testing specificity of higher levels of slippage to the viral polymerase, these experiments also put upper bounds on slippage introduced during reverse transcription." on lines 235-236. We used two host mRNAs containing GA_6G sequences (as at the TuMV *pipo* shift site), and a TuMV RdRp-knockout mutant (deltaGDD) inserted into plant cell DNA via agroinfiltration, to put upper bounds on slippage occurring during reverse transcription.

4. Would the P3NPIPO encoding genomes (i.e. with the extra A) that were detected in virus particles be viable? That is, how essential is the full-length P3 protein for infections? The ~2% trade-off is probably negligible when weighted against the need to express the transframe protein. However, the low level of slippage observed could be, in part, the consequence of selection to minimize the production of such defective genomes. This may be worth mentioning.

Upon entering a cell, P3NPIPO-encoding genomes (i.e. with the extra 'A') would express P1-HCPro-P3NPIPO but not the full-length polyprotein P1-HCPro-P3-6K1-CI-6K2-NIaVPg-NIaPro-NIb-CP. Without expression of the viral RdRp, there would not even be opportunity to restore a normal viral genome via transcriptional deletion of the inserted A. It is conceivable that *translational* +1/-2 frameshifting on the GA_7 sequence could result in expression of minute amounts of RdRp and restoration of infectivity. However, for all intents and purposes, we expected that edited genomes may be regarded as non-viable unless supported by a helper genome.

The reviewer makes a good point that the low level of slippage at the *pipo* slip site (~2%) may partly be a consequence of selection to minimize production of non-infectious genomes. However, this topic needs to be discussed in the context of the mean (non-programmed) insertional error rate of the viral polymerase, which, from the whole-genome sequencing data added in response to Reviewer 3, is of order 0.001% per nucleotide, i.e. around 0.1 insertions per genome. This means that, while ~2% of genomes are non-infectious due to insertions at the *pipo* site, ~10% of genomes are non-infectious due to spurious insertions at other sites. It would seem therefore that the virus could tolerate somewhat higher levels of (programmed) slippage at the *pipo* site (e.g. by having a more slip-prone sequence, such as a longer homopolymeric run) without greatly changing the proportion of non-infectious to infectious genomes, and thus selection to minimize production of non-infectious genomes is perhaps unlikely to be the key reason for the low level of *pipo* slippage. In the interests of brevity and focus, we think that these discussions are best left out of this manuscript.

5. Could minus strand be analyzed to help determine if the slippage occurs during plus or minus strand synthesis?

We have made attempts to analyze slippage in minus-strand RNA. In this analysis we saw that the fraction of minus-strand sequences with insertions was ~50-75% of that detected for positive-strand RNA. However the total amount of minus-strand RNA relative to positive-strand RNA is very low and we have noticed the undesired potential for mis-priming and self-priming from positive-strand RNA during negative-strand specific reverse transcription. This, combined with the low efficiency of slippage, means that the results of the minus-strand analysis are unreliable. Minus strand coverage in the whole genome sequencing data is also too low to reliably quantify slippage.

We have also compared slippage efficiency (sequencing of the positive sense) on the WT slip site and a reverse-complemented slip site, fused into a different genomic location where they do not affect virus viability. Although these results (one round of high throughput sequencing so far) are consistent with the result of the previous paragraph, it is possible that positive-strand synthesis is fundamentally different from reverse-strand synthesis with regards to the efficiency of transcriptional slippage (since reverse-strand synthesis is thought to use a single-stranded template, whereas positive-strand synthesis may involve displacement of a previously synthesised positive-strand).

© European Molecular Biology Organization

For these reasons, we feel that it is premature to include our attempts to discriminate slippage during positive-sense and negative-sense synthesis, and we feel that a proper assessment of this question is beyond the scope of this short report.

6. This reviewer has also read the very recently published (April 15th) short communication (i.e. Letter to the Editor) on this subject by the Garcia lab in Journal of Virology. This article and the current manuscript both provide data supporting a slippage mechanism in potyviruses. However, the current manuscript provides a much more comprehensive study that, importantly, includes in planta experiments that support the biological relevance of this phenomenon. Consequently, this work will be widely recognized as an important contribution in this area of research.

7. The discussion, though interesting, seems a bit drawn out - and thus would benefit from a bit of judicious trimming.

We have shortened and streamlined the Discussion.

Referee #2:

The Potyviridae family of viruses is comprised of positive-sense, single-stranded RNA viruses that are infectious to many plant species and are responsible for more than half of viral crop damage globally. Recently, a conserved overlapping gene in the -1/+2 frame designated as pipo has been identified in the P3-encoding region of the viral polyprotein coding sequence. PIPO is expressed as a fusion protein with the N-terminus of P3, and is essential for virus cell-to-cell transmission. While the mechanism underlying the expression of PIPO was previously suggested to be via a programmed frameshifting mechanism, the current work demonstrates that PIPO expression occurs via transcriptional slippage by the viral RNA polymerase, which generates a minor subset of viral transcripts that contain an additional adenosine insertion within a conserved GAAAAAA sequence. Mutations that inhibit transcriptional slippage or prevent expression of PIPO prevented viral spread to the upper leaves following biolistic inoculation of Nicotiana benthamiana plants. High throughput sequencing indicated a small enrichment of a single adenosine insertion event in WT, M1 and M2-infected samples compared to the P and FSko-infected samples, and similarly in purified virions and polysome fractions, in support of transcriptional slippage as the mechanism for PIPO expression. Overall, the finding is significant and resolves the mechanism of pipo expression in this virus. The results are convincing and the conclusions are supported by the results. I only have minor comments that improve or clarify their conclusions.

Comments:

1. On Page 5, lines 127-128, a statement was made that "The signal strengths and expression did not change further during later stages of infection (7 to 21 d.p.i)." A western blot showing a time point late in infection (ie. 21 d.p.i) should be included in Fig. 2 to support this.

We have added a reference to Fig 3C (previously Fig 4B) which now shows both P3 and P3N-PIPO (anti-V5 WB) at 21 d.p.i.. The text now reads "... P3N-PIPO became detectable in minute quantities only at later timepoints (around 6 d.p.i.; see also Fig 3C for 21 d.p.i.)" (lines 122-124). The specific reference to "signal strengths" has been deleted during manuscript shortening and in view of Reviewer 3's comment about lack of precise quantitation in these data.

2. In Fig. 4b, it would be informative to also include the western blot for anti-V5/P3N-PIPO.

We have extended the anti-V5 WB to include P3N-PIPO. (Now renumbered as Fig 3C.)

4. Do M1 and M2 mutations affect viral titer/infectivity, which may suggest a minor contribution of -1 PRF for PIPO expression?

We have not seen any convincing phenotypic differences between WT and the M1 and M2 mutants. Moreover we have not seen reversion to WT. Of course it is difficult to accurately quantify small differences in plants compared to, for example, animal cell culture where one can establish

synchronous uniform infections. As discussed (lines 176-178), the lack of any convincing difference indicates that -1 PRF is either not used or not required in TuMV infection. Unfortunately we would not be able to assess minor differences via e.g. competition assays or extended serial passage since mutations adjacent to the shift site may also slightly modify transcriptional slippage efficiency, or potentially also polymerase progressivity at the slip site if the conserved 5' 'G' is relevant to this.

5. From Fig. 5, it is difficult to quantitatively assess if the mutants have defects in replication based on RT-PCR. Northern blot or real-time RT-PCR should be performed and a normalizing control (other than EtBr staining of rRNA) should be used.

(Now renumbered as Fig 4). The purpose of these data is simply to show that the mutants do replicate (in case the GFP signal observed in single cells were coming from translation of RNA transcribed directly from input plasmid). Precise quantification is not essential here. Given that the mutants are replicating within single cells, whether at WT levels or potentially (but unlikely) at a reduced level, the lack of a movement phenotype indicates that P3N-PIPO expression is either inhibited or prevented in these mutants. Undoubtedly, there are quantitative differences in viral RNA and protein between mutant and WT (note the RuBP-L loading control is fainter in WT) because the P and FSko mutants are restricted to single cells, whereas WT spreads through many cells (also synchronous versus asynchronous infection). Under this background, we think that northern blot or real-time RT-PCR would not provide any more accurate quantification of viral replication or additional value to the claims. These issues are dealt with in lines 180-200 (line numbering as per revised version). We have also modified "the mutants unable to move from cell-to-cell (i.e. P and FSko) do not have significant impairment in replication or accumulation" to "the mutants unable to move from cell-to-cell (P and FSko) are still able to replicate and accumulate" (lines 197-198).

Referee #3:

SUMMARY
In this manuscript Olspert et al. analyse the molecular mechanism that leads to the expression of the P3N-PIPO gene product in the Potyviridae. The authors attempt to unravel whether a translational or a transcriptional frame shifting is responsible for the production of this alternative protein. The authors engineer viruses expressing GFP and encoding a N-terminal-V5-tagged P3 and P3N-PIPO proteins and first demonstrate that systemic leaves of infected plants express both proteins, the latter accounting for about 2% of total. Then the authors generate point mutant viruses, which would impede ribosomal frame shifting, transcriptional frame shifting or both and analyse the expression of GFP in systemic leaves of infected plants. Results show that mutants affecting transcriptional frame shifting are affected in GFP expression but mutants affecting ribosomal frame shifting are not. As a control, the authors show that replication and expression of all these mutant viruses in agro-infected cells is normal. Finally, the authors carry out deep sequencing of the frame-shifting region of the genome from virus infected cellular RNA and virion RNA and show that only mutations affecting transcriptional frame shifting abolish the insertion of a single A residue at the frame shifting point.

GENERAL COMMENTS
The manuscript is well written (but see below) and the figures are clear. The experiments are well performed and controlled. The results clearly support the conclusions put forward. I include below some comments and suggestions for improvement.

SPECIFIC POINTS
1. Results. Lines 131-134.
The experiments do not tell about the requirement of P3N-PIPO.

We wrote "Nonetheless these experiments demonstrated that P3N-PIPO is produced and required only in very small amounts ... [relative to P3]". Since P3N-PIPO has already been shown to be essential (Chung et al., 2008), and our new data shows that WT TuMV only produces very small amounts of P3N-PIPO, we draw the conclusion that only small amounts of P3N-PIPO are required. Nonetheless we have taken the reviewer's point on board and rewritten this as "Nonetheless these

experiments demonstrated that P3N-PIPO is produced only in very small amounts ... [relative to P3]" (lines 127-128).

If the levels of expression of P3N-PIPO were so small that efficiency of frame shifting cannot be determined, a change in the proportion of P3/P3N-PIPO would not be detectable either.

While we might be unable to detect say a 1.5-fold change, we would certainly be able to detect say a 5-fold change. The statement "At no early timepoint, i.e. when virus is moving rapidly from cell-to-cell, was there any detectable increase in the ratio of P3N-PIPO to P3." was simply meant to indicate that we searched for but did not observe *qualitative* differences in P3N-PIPO expression at different timepoints. Nonetheless, taking the reviewer's comment on board, and also with a view to brevity, we have now deleted this statement.

2. Results. Section on deep sequencing.
It would have been interesting to perform whole-genome deep sequencing of virus infected cells and virions to test whether other positions in the viral RNA show insertions or deletions in a proportion similar to that detected in the GA6 site.

We have now performed whole-genome sequencing of total RNA and virion RNA and added a new figure (new Supplementary Fig S1) showing total coverage of the TuMV genome, and the positions and numbers of single-nucleotide insertions. These data nicely show that insertions occur at a much higher level at the *pipo* GA_6 slip site (~2% of all reads, as before) than anywhere else in the TuMV genome.

3. Results. Lines 279-322.
The description of results in this section is rather cumbersome. It could be simplified to make it shorter and more amenable to the readers.

We have greatly shortened this section focusing on the essentials for the manuscript text, with supporting details moved to Supplementary Results, and bioinformatic details moved to Supplementary Methods.

4. Discussion.
Discussion is unnecessarily long and at some points reiterative. It could be pruned and simplified.

We have shortened and streamlined the Discussion.

5. Discussion. Lines 401-403
The role of the 5'-G is discussed but it could have been tested experimentally.

We have shortened and streamlined the Discussion of the 5' G. Experimentally testing the role of the 5' G requires construction, plant infection, and high-throughput sequencing of a large number of virus mutants, since the effect of the G is likely to be modulated by the flanking nucleotides (e.g. propensity for stronger G:C versus weaker A:U base-pairings); further, in TuMV, where the conserved GA_6 is preceded by an additional 'G', it would be important to assess both 'G's. This requires copying the sequence to a different genomic location where it can be freely mutated without affecting P3/P3N-PIPO expression or amino acid sequence. Moreover, we need to properly discriminate slippage during negative-sense and positive-sense synthesis (see response to Reviewer 1). Finally, the effect of the 'G' may not be on slippage efficiency *per se*, but perhaps on avoiding polymerase stalling or other effects which are not easy to accurately measure for the low-efficiency events occurring at the *pipo* shift site. We are pursuing this work, but we feel that the large number of mutants to be tested are beyond the scope of the current short report.

MINOR POINT
1. Introduction. Line 73. DownStream.

Spelling error corrected.

2nd Editorial Decision 03 June 2015

We have now received the comments from referees 1 and 2, who assessed your revised manuscript. As you will see from the reports below, the referees support its publication in EMBO reports with no further comments. I am therefore writing with an 'accept in principle' decision, which means that I will be happy to accept your manuscript for publication once the study has been adapted to EMBO reports Scientifc Report format and a few minor issues have been addressed, as follows.

- Our scientific reports do not include a separate conclusions section, but discuss the findings within the "Results & Discussion" section wherever appropriate. They cannot include supplementary results either. Please go through your text once more and ensure that all relevant information is provided within the main text, and the conclusions are integrated into the "Results and Discussion" section.

- The data accessibility part of the Materials and Methods needs to be included in the main text.

After all remaining corrections have been attended to, you will receive an official decision letter from the journal accepting your manuscript for publication in the next available issue of EMBO reports. This letter will also include details of the further steps you need to take for the prompt inclusion of your manuscript in our next available issue.

Thank you for your contribution to EMBO reports.

REFEREE REPORTS:

Referee #1:

In this reviewer's opinion, the authors have adequately addressed the comments of this reviewer as well as the comments of the other two reviewers.

Referee #2:

The author has addressed the comments appropriately and added results (Fig. 3C) that improve the clarity of the manuscript.

2nd Revision - authors' response 05 June 2015

Thank you very much for your email.
I have just resubmitted the manuscript with the requested modifications.

3rd Editorial Decision 08 June 2015

I am very pleased to accept your manuscript for publication in the next available issue of EMBO reports. We will also fast-track online publication as much as possible. Thank you for your contribution to our journal.

I have commissioned a highlight of your study and the related J. Viology letter to the editor from Andrew White, which will appear in the same issue of the journal.

Thank you again for your contribution to EMBO reports and congratulations on a successful publication.

© European Molecular Biology Organization
